# SigAlign: an alignment algorithm guided by explicit similarity criteria

**DOI:** 10.1093/nar/gkae607

**Published:** 2024-07-16

**Authors:** Kunhyung Bahk, Joohon Sung

**Affiliations:** Interdisciplinary Program in Bioinformatics, College of Natural Sciences, Seoul National University, 1 Gwanak-ro, Gwanak-gu, Seoul 08826, Korea; Interdisciplinary Program in Bioinformatics, College of Natural Sciences, Seoul National University, 1 Gwanak-ro, Gwanak-gu, Seoul 08826, Korea; Genome and Health Big Data Laboratory, Graduate School of Public Health, Seoul National University, 1 Gwanak-ro, Gwanak-gu, Seoul 08826, Korea

## Abstract

In biological sequence alignment, prevailing heuristic aligners achieve high-throughput by several approximation techniques, but at the cost of sacrificing the clarity of output criteria and creating complex parameter spaces. To surmount these challenges, we introduce ‘SigAlign’, a novel alignment algorithm that employs two explicit cutoffs for the results: minimum length and maximum penalty per length, alongside three affine gap penalties. Comparative analyses of SigAlign against leading database search tools (BLASTn, MMseqs2) and read mappers (BWA-MEM, bowtie2, HISAT2, minimap2) highlight its performance in read mapping and database searches. Our research demonstrates that SigAlign not only provides high sensitivity with a non-heuristic approach, but also surpasses the throughput of existing heuristic aligners, particularly for high-accuracy reads or genomes with few repetitive regions. As an open-source library, SigAlign is poised to become a foundational component to provide a transparent and customizable alignment process to new analytical algorithms, tools and pipelines in bioinformatics.

## Graphical Abstract

**Graphical Abstract:**
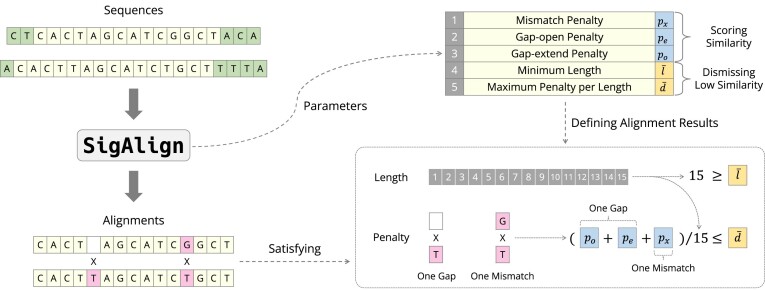

## Introduction

In bioinformatics, sequence alignment is a fundamental process for identifying similar regions between sequences, often serving as the first but computationally intensive step in analytical tasks. Sequence alignment algorithms are categorized into non-heuristic and heuristic aligners. Non-heuristic aligners, such as dynamic programming methods (1,2), precisely calculate the penalty score between sequences to identify the alignment with the lowest penalty, which is considered the global optimum and recognized as the computational gold standard (3). However, non-heuristic aligners face an inherent speed limitation. Despite recent advancements in algorithmic efficiency (4) and hardware acceleration (5), they still operate in quadratic time relative to sequence length in the worst case. Consequently, heuristic aligners, employing approximation methods for high throughput, have become essential for processing large volumes of data. Although these aligners do not guarantee the identification of the global optimum alignment, they have demonstrated adequate accuracy for domain-specific problems. Tools such as BWA-MEM (6) and bowtie2 (7) for read mapping, alongside BLASTn (8) for database searches, are prime examples of heuristic aligners that have been widely adopted in research applications.

Heuristics in sequence alignment commonly employ the ‘seed-and-extend’ method (9), which selects an initial point for alignment (seeding) and expands the alignment from this point (extending), rather than computing alignment scores across the entire sequence range. For example, this ‘seed-and-extend’ approach can initiate alignment at a matching 12-mer and cease extension when the penalty score reaches 50. The implementation of ‘seed-and-extend’ varies among heuristic aligners (9). Specifically, BWA-MEM identifies the longest, non-overlapping *k*-mer (SMEM) as the starting point for alignment (6), while bowtie2 prioritizes alignment at the highest-scoring *k*-mer (7). Furthermore, the data structures utilized to pinpoint these starting points differ, with BWA-MEM and bowtie2 using the FM-index (10), and BLASTn employing a hash table (8). Besides ‘seed-and-extend’, heuristic aligners incorporate distinct heuristic steps (11). For example, bowtie2 outputs an alignment even if not all seeds have been extended, provided the alignment’s penalty exceeds the best or second-best penalty multiple times consecutively (7). Overall, heuristic aligners achieve high throughput and satisfactory accuracy for the intended research domain through various heuristic techniques, many of which are unique to specific aligners.

However, the reliance on heuristics for alignment results introduces two primary challenges. First, the criteria for determining which alignment results are included or excluded are not transparent. For instance, there’s a misconception that BLASTn includes all alignments that meet its ‘E-value’ threshold for statistical significance (12,13), yet some alignments may be omitted if they fail to clear heuristic filters applied in prior stages (13). Similarly, within the SAM format, the Mapping Quality (MAPQ) serves as a measure of the correctness of alignment (14), and is crucial filtering criteria for subsequent variant calling steps (15,16). Nonetheless, alignments with a MAPQ lower than the reported value might be excluded (17), and the methodology for calculating MAPQ is often not transparently disclosed in the literature or manuals of aligners (18). These instances of *E*-value and MAPQ exemplify the opaque nature of outputs generated by tool-specific heuristics, lacking clear cutoffs.

The complexity of heuristic aligners’ parameter space presents a second major challenge. For example, BLASTn and BWA-MEM include 22 and 15 parameters that influence results, respectively (Supplementary Table S1). The interaction among certain parameters makes their effects on outcomes non-linear and difficult to predict (19). As a result, fully optimizing this extensive range of parameters is a daunting task, often cited as a performance optimization hurdle for heuristic aligners (19–21). Although these aligners are designed to perform effectively with default settings, applying heuristic aligners to different research domain frequently requires parameter adjustments. Tailored parameter settings are known to improve performance, particularly in specialized applications like multiple bacterial sequence alignments (21,22). Nevertheless, even in well-adapted metagenome analysis workflows (23,24), alignment algorithms still operate under default settings or with minimal adjustments (e.g., ‘–very-sensitive’ for bowtie2), underscoring the need for better optimization strategies for specific research purposes.

The reliance on the ‘seed-and-extend’ strategy is crucial for attaining the necessary processing speeds in sequence alignment. Nonetheless, we argue that the primary shortcomings of heuristic aligners stem not from the ‘seed-and-extend’ approach itself, but rather from their reliance on heuristics for result selection. To overcome these limitations, we suggest defining foundational cutoffs for the alignment results. These cutoffs then guide the entire alignment process. We aim to replicate the rapid processing of heuristic aligners while basing the results on a simpler and more transparent parameter set.

Building on this concept, we introduce SigAlign (Similarity-guided Alignment Algorithm), a novel alignment algorithm and its corresponding software library. SigAlign utilizes the affine gap penalty scheme, widely accepted in DNA/RNA alignment, alongside two specific cutoffs: minimum length (MinL) and maximum penalty per length (MaxP). These cutoffs are grounded in biological rationale that longer alignments with lower penalties are improbable to arise by chance. SigAlign shares the ‘seed-and-extend’ approach, but unlike heuristic aligners, it implements exact cutoffs to guide the entire alignment process and outcome determination. Notably, SigAlign simplifies user interaction by requiring adjustment of only five parameters—three related to the affine gap penalties and two serving as cutoffs. This approach allows users to effectively steer the alignment process by modifying parameters that have direct biological relevance.

This paper elucidates the SigAlign algorithm, specifically addressing the computation of derivatives essential for the ‘seed-and-extend’ process, utilizing the five input parameters. Moreover, our study evaluates SigAlign’s efficacy relative to traditional heuristic aligners by examining both speed and accuracy metrics. Through comparative testing, we delineate SigAlign’s strengths and propose its areas of utility in bioinformatics research. We foresee SigAlign, as an open-source library, becoming an integral component of bioinformatics workflows, offering a clear and efficient approach to sequence alignment.

## Materials and methods

### Algorithm overview

#### Definition of inputs and outputs

SigAlign operates with two inputs: sequences and parameters. Sequences, which can be any single-byte character such as ASCII code, are categorized into query and target. While this ‘Materials and methods’ section is dedicated to the alignment of a single query with a single target, SigAlign, in its practical implementation, is designed to align a single query against a ‘reference’—a collection of numerous target sequences. The parameters consist of affine gap penalties—mismatch, gap-open, and gap-extend—and cutoffs defined by the minimum length (MinL) and maximum penalty per length (MaxP). While not utilized in this paper, SigAlign also incorporates an optional match bonus that is sometimes applied to counteract the penalties, in addition to three affine gap penalties (for more details, see Supplementary Note 1).

The alignment output includes (i) the length of alignment, (ii) the penalty value, (iii) the position (start/end indices of sequences) and (iv) an operation vector, consisting of matches, substitutions, insertions, or deletions. If no alignment meets the cutoffs of MinL and MaxP, SigAlign reports no results. When multiple alignments satisfy the cutoffs, SigAlign prioritizes the alignment with the longer length. If the alignments have the same length, the one with the smaller penalty is prioritized. Additionally, SigAlign may exclude an alignment from the results if its position overlaps with that of a higher-priority alignment (see Supplementary Note 2 for a detailed definition of output).

Notably, the output of SigAlign is completely reproduced using dynamic programming methods, such as the Needleman-Wunsch algorithm (1), which performs alignment employing the same affine gap penalty and subsequently filters the results using the same cutoff criteria. See Supplementary Note 3 for the detailed process of this replication.

#### Overall workflows

**Figure 1.**
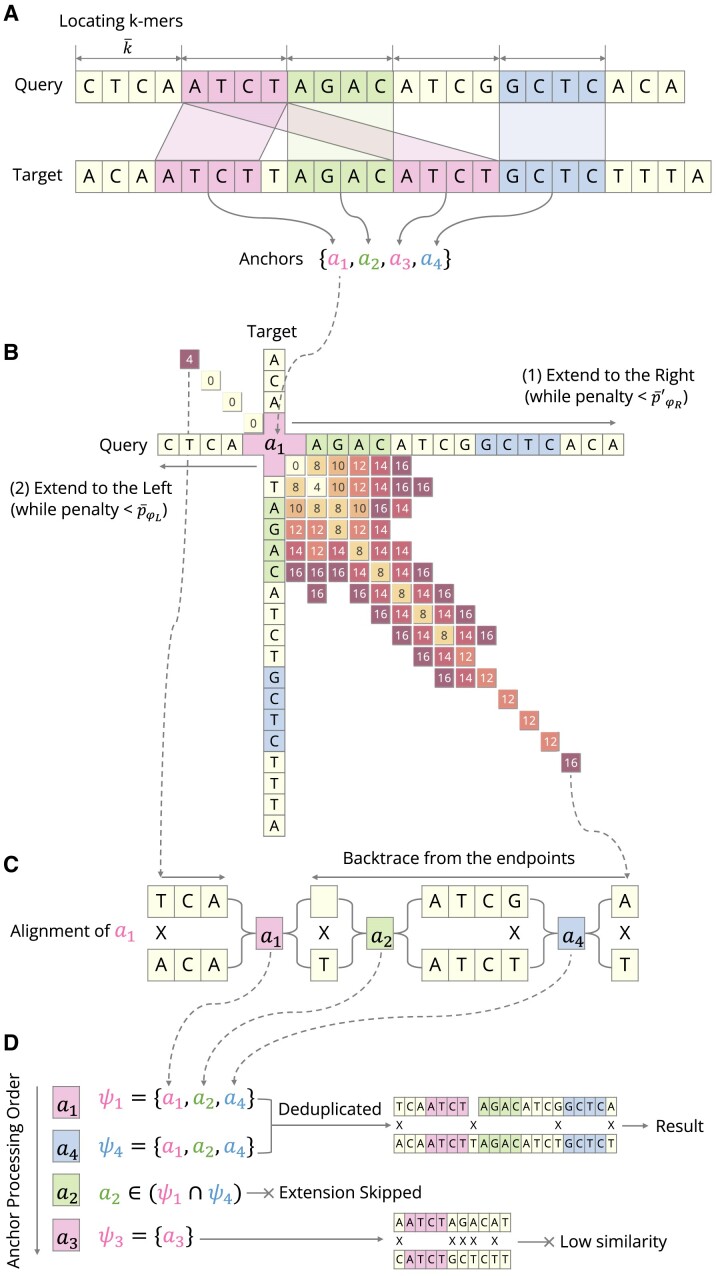
Workflows of algorithm. (**A**) Illustrates the identification of anchors, which serve as alignment starting points, derived from matching *k*-mers between the ‘Query’ and ‘Target’ sequences. K-mers are defined as consecutive, same-sized sequences (denoted as $\bar{k}$ in this paper). (**B**) Demonstrates the extension process from a given anchor (with *a*_1_ as an example in the figure). The matrix represents the accumulation of penalties during the alignment process. Initially, the alignment extends to the right until the penalty ${\bar{p}\prime }_{\varphi _R}$ (reduced right spare penalty) is reached. Subsequently, the alignment extends to the left until the ${\bar{p}}_{\varphi _L}$ (left spare penalty) is reached. (**C**) Depicts the backtracing step, which is employed to extract the alignment from the extended end-points. During backtracing, all traversed anchors (such as *a*_2_ and *a*_4_) are identified. An ‘X’ denotes a mismatch or gap in the alignment. (**D**) Details the evaluation step using anchor symbols (ψ). Redundant alignments are removed, and those that do not meet the similarity cutoffs (MinL and MaxP) are discarded. It’s noteworthy that the extension step for anchor *a*_2_ is skipped in the process.

Figure 1 depicts the comprehensive workflow of SigAlign, which is structured around three principal steps focused on the ‘anchor’ —the alignment’s initiation point. These steps underscore the methodical approach from the preliminary identification of alignment candidates to the conclusive alignment determination, guided by the parsimonious parameters of SigAlign.

1. Determining anchors: The alignment starting points are identified by dividing the query into consecutive patterns, and then locating exact matches in the target (Figure 1A).
2. Extending anchors: From these starting points, the alignments extend in both directions until they meet a specified penalty threshold (Figure 1B).
3. Evaluating anchors: Each alignment is verified against the predetermined cutoffs for reporting (Figure 1C, D).

Given the non-heuristic nature of SigAlign, we elaborated on the calculation methods of variables and their derivation process in the subsequent ‘Detailed Algorithm Workflows’ subsection for each step.

#### Core technical differences

SigAlign employs the ‘seed-and-extend’ strategy, a common approach used by most heuristic aligners, which involves identifying the starting point of an alignment and extending from that position (9). SigAlign’s distinctions in algorithms and operation are described in three aspects: (i) algorithmic advancement within the ‘seed-and-extend’ approaches; (ii) other components of alignment algorithms unique to SigAlign; (iii) advancements in software implementation.

In the ‘seed-and-extend’ process, SigAlign selects exactly matched *k*-mers as alignment starting points, termed ‘anchors’ in this paper, and extends these anchors until reaching a specific penalty score. This approach shares similarities with other heuristic aligners; for example, SigAlign utilizes the FM-index (10), based on the Burrows-Wheeler Transform (25), for anchor identification, similar to BWA-MEM (6) and bowtie2 (7). The method of sliding *k*-mers to find matches resembles that of bowtie2 and Novoalign (http://novocraft.com/), while halting the alignment at a specific penalty is similar to BLASTn (8) and BWA-MEM. However, while other aligners handle both k-mer size and alignment stop penalty as static input parameters, SigAlign dynamically calculates these two variables within the algorithm. For instance, adjusting the gap affine penalty values in SigAlign affects the k-mer size, and the alignment stop penalty may vary depending on the anchor’s position. Details on these dynamic calculations are provided in Steps 1 and 2 of the ‘Detailed algorithm workflows’ section.

Beyond the ‘seed-and-extend’ process, SigAlign employs a unique step for deduplicating results. Unlike most heuristic aligners that select only a subset of high-priority anchors, SigAlign performs alignments for all anchors to output all alignments that satisfy the cutoffs. Consequently, SigAlign’s anchors are more likely to produce identical results compared to other aligners. To avoid outputting duplicate results, SigAlign assigns hash values, called ‘symbols’, to each alignment result for deduplication. The use of symbols accelerates the deduplication process and helps skip anchor alignments under specific conditions. A detailed discussion of this process is provided in Step 3 of the ‘Detailed Algorithm Workflows’ section.

Apart from algorithmic differences, SigAlign also exhibits distinctions in software implementation. These implementation distinctions, pertaining to performance optimization or flexibility, arise from variations at the source code level rather than the algorithm itself. For instance, SigAlign employs an FM-index (10) with a lookup table (26) for determining the starting position of alignments (Step 1). While existing papers (26) only support nucleotide or amino acid sequences, SigAlign supports all single-byte characters, and the memory space decreases with fewer characters used. Furthermore, by not utilizing processor-specific instructions (e.g. SSE, AVX), SigAlign can operate on web browsers that support Web Assembly. These software implementation distinctions are more comprehensively detailed in Supplementary Note 4.

### Detailed algorithm workflows

This section outlines the algorithm’s process in a sequential manner. Initially, each stage is introduced by defining its purpose, followed by a description of the expected output, and an exploration of associated considerations and challenges. Subsequently, we detail the approach to ascertain specific values pertinent to that stage.

In this section about detailed algorithm workflows, we employ terms with specific definitions for what have traditionally been referred to as ‘alignment.’ While ‘alignment’ conventionally encompasses both the process of matching similar sequences and the resultant data, here, ‘alignment’ pertains solely to the output data, and ‘algorithm’ designates the process to achieve those results. In this context, an alignment can be considered as a vector of operations with its starting point. Each operation defines the relationship between the query and target sequences, as one of four types: Match, Substitution, Insertion and Deletion. The starting point is represented by the index pair of query and target sequences. The length of the alignment is identical to the length of the vector of operations.

We adopt specific notational conventions for various elements related to penalty, length, and penalty per length. Here, *p* represents penalty, *l* pertains to length, and *d* (short for distance) signifies penalty per length. We designate the mismatch, gap-open, and gap-extend penalties as *p*_*x*_, *p*_*o*_, and *p*_*e*_, respectively. Our similarity cutoffs—MinL and MaxP—are denoted as $\bar{l}$ and $\bar{d}$.

#### Determining anchors (Step 1)

The initial step involves identifying the alignment’s starting points, termed as ‘anchors’. This is accomplished in two phases: (i) dividing the query sequence into consecutive *k*-mers and (ii) locating an exact match for each *k*-mer within the target sequence. Figure 1A visually represents this step. The mechanics of this step bear resemblance to a quotient filter (or a bloom filter). If a matching alignment exists under the given cutoffs, at least one anchor will emerge. In the absence of any anchor, it is inferred that no satisfying alignment exists. For locating all positions of the k-mers, SigAlign employs the FM-index (10), which contains a compressed index data structure containing the Burrows-Wheeler transformed text (25). Given the computational intensity of FM-index operations (27), we have adopted a strategy that sets the sliding window size equal to the k-mer size to reduce the number of operations. This method diverges from the conventional sliding window approach, such as *q*-grams (28), aiming to minimize FM-index utilization. Central to this step is the selection of the maximal k-mer within the operational range of the quotient filter, a data structure that ensures the existence of at least one anchor if an alignment satisfying the cutoffs exists, thereby minimizing the required number of anchors. Fewer anchors translate to lower computational costs in anchor extension in the subsequent Step 2.

Let’s denote the size of a *k*-mer as *k*. Initially, we determine the range of the length $\hat{l}_n$ of an alignment that can be represented by *nk*-mers. The length $\hat{l}_n$ is composed of the length of the query $l^{\prime }_n$ included in the alignment and the total number of gaps (Deletion) *g*_*n*_, expressed as $\hat{l}_n = l^{\prime }_n + g_n$. Considering a maximum residue length of *k* − 1 at both ends of the *k*-mer block, $l^{\prime }_n$ falls within the range [*nk*, *nk* + 2(*k* − 1)]. Consequently, the range of $\hat{l}_n$ is given by:


(1) \begin{equation*} \hat{l}_n \in \left[ nk + g_n, nk + 2(k - 1) + g_n \right]. \end{equation*}

Next, we calculate the penalty $\hat{p}_n$ for alignments where none of the *n* k-mers match. We treat $\hat{p}_n$ as a recursive formula, adding penalties progressively to $\hat{p}_{n-1}$. For a single *k*-mer (*n* = 1), the minimum $\hat{p}_1$ corresponds to the penalty for one mismatch or one gap. However, for two *k*-mers (*n* = 2), as they can share consecutive gaps, $\hat{p}_2$ requires separate calculation from $\hat{p}_1$. We therefore divide the calculation of $\hat{p}_n$ based on the parity of *n* ($m \in \mathbb {Z}^{+}$, $\hat{p}_0 = 0$):


\begin{eqnarray*} \hat{p}_{2m} &\ge &\hat{p}_{2m-2} + \min \left( 2p_x, p_o+2p_e \right) \\ \hat{p}_{2m-1} &\ge &\hat{p}_{2m-2} + \min \left( p_x, p_o+p_e \right). \end{eqnarray*}

To streamline this, we define constants *p*_1_ and *p*_2_ for odd and even *n* values respectively:


\begin{eqnarray*} p_1 &=& \min \left( p_x, p_o+p_e \right) \\ p_2 &=& \min \left( 2p_x, p_o+2p_e \right) - p_1. \end{eqnarray*}

Consequently, $\hat{p}_n$ is formulated as:


(2) \begin{equation*} \hat{p}_n \ge p_1 \left\lceil n / 2 \right\rceil + p_2 \left\lfloor n / 2 \right\rfloor . \end{equation*}

(Refer to Supplementary Figure S1 for a visual explanation of this induction process.)

Next, we calculate the penalty per length of alignment $\hat{d}_{n}$ when none of the *nk*-mers match the target. Initially, we consider the range of $\hat{l}_n$. While theoretically, *g*_*n*_ can be infinitely large, imposing no upper limit on $\hat{l}_n$ (Equation 1), practical considerations in Equation 2 suggest a limitation. Specifically, when $\hat{p}_n$ is minimized, the number of gaps per k-mer is capped at 1, subject to the penalties (*p*_*x*_, *p*_*o*_, *p*_*e*_). Hence, for *g*_*n*_ ≤ *n*, $\hat{p}_n$ can equal its minimum, but for *g*_*n*_ > *n*, $\hat{p}_n$ increases by *p*_*e*_ for each additional gap (*g*_*n*_ − *n*) (refer to Supplementary Figure S2 for the relationship graph between $\hat{l}_n$ and $\hat{p}_n$). To manage the variability in $\hat{p}_n$ based on $l^{\prime }_n$’s range, we confine the maximum range of $\hat{l}_n$ to cases where $\hat{p}_n = \min \hat{p}_n$. Given the increment in *g*_*n*_ is only up to the point where $\hat{p}_n = \hat{p}_{n+1}$, the maximum *g*_*n*_ is $n+(\hat{p}_{n+1}-\hat{p}_{n})/p_e$. Since the difference between $\hat{p}_{n+1}$ and $\hat{p}_{n}$ depends on *n*’s parity, and is either *p*_1_ or *p*_2_, with *p*_1_ typically greater or equal to *p*_2_, we use *p*_1_ to limit *g*_*n*_’s range: *g*_*n*_ ∈ [0, *n* + *p*_1_/*p*_*e*_]. Therefore, with Equations (1) and (2), the range of $\hat{d}_{n}$ is calculated as follows:


(3) \begin{equation*} \hat{d}_{n} \ge \frac{\min \hat{p}_{n}}{\max (\hat{l}_{n} | \hat{p}_n = \min \hat{p}_{n})} = \frac{p_1 \left\lceil n / 2 \right\rceil + p_2 \left\lfloor n / 2 \right\rfloor }{(n+2)(k+1)-4+p_1/p_e},\nonumber\\ \end{equation*}

when $\hat{l}_n \in \left[ nk , {(n+2)(k+1)-4+p_1/p_e} \right]$. Separating the cases for odd and even *n* to streamline Equation (3), and removing the floor and ceiling functions, we obtain:


(4) \begin{equation*} \begin{split} \hat{d}_{2m} &\ge \frac{m(p_1+p_2)}{(2m+2)(k+1)-4+p_1/p_e} \\ \hat{d}_{2m-1} &\ge \frac{m(p_1+p_2)-p_2}{(2m+1)(k+1)-4+p_1/p_e}. \end{split} \end{equation*}

Let’s revisit our objective to ensure ‘no alignments that meet the cutoffs exist in the absence of any matching *k*-mers’. *k* must be set such that for all *n* with $\hat{l}_n \ge \bar{l}$, $\hat{d}_{n}$ is always greater than $\bar{d}$: $\forall n : \hat{l}_n \ge \bar{l} \rightarrow \hat{d}_{n} >\bar{d}$. When $\hat{n}$ denotes the smallest *n* that makes $\hat{l}_n$’s maximum value equal to or greater than $\bar{l}$, $\hat{n}$ is defined as:


\begin{eqnarray*} \hat{n} = \left\lfloor \frac{\bar{l}+4-p_1/p_e}{k+1} \right\rfloor -2. \end{eqnarray*}

In Equation (4), although the relationship between $\hat{d}_{2m}$ and $\hat{d}_{2m-1}$ is not fixed, $\hat{d}_{2m+1}$ and $\hat{d}_{2m+2}$ are consistently greater than $\hat{d}_{2m}$ (refer to Supplementary Note 5 for detailed calculation). Therefore, it suffices to check if $\hat{d}_{n}$ is greater than $\bar{d}$ for $n = \hat{n}$ and $\hat{n}+1$. Finally, the maximal pattern size $\bar{k}$ can be determined as:


\begin{eqnarray*} \bar{k}=\max \left\lbrace k\in \mathbb {Z}_+: (\min \hat{d}_{\hat{n}}> \bar{d}) \wedge (\min \hat{d}_{\hat{n}+1}> \bar{d}) \right\rbrace . \end{eqnarray*}

Since $\hat{d}_{n}$ is a non-differentiable discontinuous function and the search space is small, we used an iterative approach to identify the largest value of *k* for each $\hat{n}$ by incrementing $\hat{n}$ starting from 1. The detailed algorithm to calculate $\bar{k}$ is described in Supplementary Note 6.

The resulting anchor is denoted through an index pairing of the query and target sequences (*Q* and *T*), as:


\begin{eqnarray*} a_n=(i_{n,Q},i_{n,T})\in (\mathbb {N}_0,\mathbb {N}_0). \end{eqnarray*}

#### Extending anchors (Step 2)

The extension of anchors involves computing penalties towards the ends of a sequence, using the anchor as a starting point. Figure 1B provides a visual representation for this step. Utilizing the non-adaptive version of the wavefront algorithm (4), which is grounded in the diagonal-transition strategy (29), enables reduced memory use and accelerated speed relative to the dynamic programming method. The result of this step is a preliminary alignment, consisting of multiple extension endpoints with their associated penalties, which subsequently translates to the final alignment during the ‘backtrace’ phase, elaborated in Step 3. Extension may terminate before reaching the sequence’s end, particularly upon meeting a predetermined ‘spare penalty’ value, to avert algorithmic slowdown and excessive memory use. In SigAlign, the spare penalty is dynamically determined, ensuring alignments consistently comply with cutoffs, contrasting with other algorithms that use a constant value for this purpose (6–8). As the real-time calculation of the spare penalty is resource-intensive, we simplified the formulas for practicality.

To begin, we define the range of the penalty value for each side. Anchor extension is executed bidirectionally: to the anchor’s left and right, denoted by $\varPhi =\lbrace \varphi _L,\varphi _R\rbrace =\lbrace \varphi ,\varphi ^\prime \rbrace$, where φ_*L*_ and φ_*R*_ represent the left and right sides, respectively, and φ′ the opposite side. The left side is defined as the direction towards smaller indices from the anchor position (*a*_*n*_), while the right side is defined as the direction towards larger indices from the anchor position. With lengths of the query (*l*_*Q*_) and target (*l*_*T*_), the residual lengths of the query and target on the right and left sides of the anchor can be defined as follows:


\begin{eqnarray*} l_{\varphi ,X\in \lbrace Q,T\rbrace }= \left\lbrace \begin{array}{l{@}{\quad}l} i_{n,X}, & \varphi =\varphi _L \\ l_X-i_{n,X}, & \varphi =\varphi _R \\ \end{array}\right.. \end{eqnarray*}

Ensuring the extension process ends when either the query or target’s end is reached, the alignment length of the side, *l*_φ_, must always be:


\begin{eqnarray*} l_\varphi \le \min (l_{\varphi ,Q},l_{\varphi ,T})+\lfloor {(p_\varphi -p_o)/p_e}\rfloor \\ \le \min (l_{\varphi ,Q},l_{\varphi ,T})+{(p_\varphi -p_o)/p_e}, \end{eqnarray*}

where *p*_φ_ ≥ *p*_*o*_. When the MaxP cutoff is satisfied ($\bar{d} \ge {(p_\varphi +p_{\varphi ^\prime })}/{(l_\varphi +l_{\varphi ^\prime })}$), the penalty value of the side, *p*_φ_, can be defined as:


(5) \begin{equation*} p_\varphi \le \frac{p_e(\bar{d}l_{\varphi ^\prime }-p_{\varphi ^\prime }) + \bar{d}p_e\min {(l_{\varphi ,Q},l_{\varphi ,T})}-\bar{d}p_o}{p_e-\bar{d}}. \end{equation*}

We denote the spare penalty for side φ as $\bar{p}_\varphi$, with $\bar{p}_\varphi \ge {p_\varphi }$. Defining the ‘penalty delta’ for the opposite side as $\Delta _{\varphi ^\prime }=\bar{d}l_{\varphi ^\prime }-p_{\varphi ^\prime }$, we determine $\bar{p}_\varphi$ by substituting $\Delta _{\varphi ^\prime }$ into Equation (5):


(6) \begin{equation*} \bar{p}_\varphi = \left\lbrace \begin{array}{@{}l@{\quad }l@{}}\left\lfloor { \Delta _{\varphi ^\prime }+\bar{d}\min (l_{\varphi , Q}, l_{\varphi , T}) } \right\rfloor , & p_\varphi < p_o \\ \left\lfloor { \frac{p_e\Delta _{\varphi ^\prime }+\bar{d}p_e\min (l_{\varphi , Q}, l_{\varphi , T})-\bar{d}p_o}{p_e-\bar{d}} } \right\rfloor , & p_\varphi \ge p_o \end{array}\right.. \end{equation*}

For Equation (6), a challenge arises in that to compute the spare penalty of one side ($\bar{p}_\varphi$), the penalty delta value on the opposite side ($\Delta _{\varphi ^\prime }$) is necessary. This implies that at least one side must calculate its spare penalty without the knowledge of the penalty delta value from the other side. We resolve this by first extending the right side of the anchor, assuming no other anchors exist on the left side. The validity of this strategy lies in the fact that even if the alignment result of the right anchor actually contains a left anchor, this result would have already been found in the alignment of the left anchor. Consequently, alignments satisfying the cutoffs cannot be missed. Given the maximum *k*-mer size ($\bar{k}$) and presuming no other anchors exist on the left, the maximum value of the left penalty delta, $\bar{\Delta }_{\varphi _L}$, can be fixed as $\bar{\Delta }_{\varphi _L}=\bar{d}(\bar{k}-1)$, as the penalty delta value for a *k*-mer without an anchor is consistently negative ($\frac{p_1}{\bar{k}} >\bar{d}$ and $\frac{p_1+p_2}{2\bar{k}} >\bar{d}$).

When calculating the right spare penalty ($\bar{p}_{\varphi _R}$) from Equation 6, we optimize the process by utilizing the *k*-mer’s reverse index (*j*), representing the index from the right in the query. This approach allows us to define the following constraint for the minimum length of the right-side sequence:


\begin{eqnarray*} \min (l_{\varphi ,Q},l_{\varphi ,T}) \le l_{\varphi ,Q} \le \bar{k}(j+2)-1. \end{eqnarray*}

In this optimization, *p*_*o*_ serves as the minimum value, eliminating the need for separate calculations when $p_{\varphi _R}< p_o$. Consequently, the simplified expression for the right spare penalty, denoted as ${\bar{p}^\prime _{\varphi _R}}$, is computed solely based on the reverse index *j*:


(7) \begin{equation*} {\bar{p}^\prime _{\varphi _R}} = f(j) = \min \left( \left\lfloor { \frac{\bar{d} \left( p_e \left( \bar{k} \left( j+3 \right) -2 \right) -p_o \right) }{p_e-\bar{d}} } \right\rfloor , p_o \right). \nonumber\\ \end{equation*}

By applying Equation (7), the right spare penalty becomes predictable via the reverse index (*j*), facilitating the pre-calculation of ${\bar{p}^\prime _{\varphi _R}}$ for any given query length. This eliminates the necessity of recalculating the right spare penalty for an identical number of k-mers in the query. Once the anchor is extended to the right, the left spare penalty ($\bar{p}_{\varphi _{L}}$) is then determined using the penalty delta from the right side ($\bar{\Delta }_{\varphi _R}$).

#### Evaluating anchors (Step 3)

The evaluation of an anchor involves extracting alignments that meet the cutoffs (MinL, MaxP) from each extended anchor. Concurrently, deduplication of results is performed by utilizing a hash symbol for each anchor, ensuring efficient processing. Notably, certain anchors are expected to produce identical results even before extension, allowing them to bypass the extension process in Step 2. This proactive approach to deduplication substantially reduces SigAlign’s computational time.

The first task is the ‘backtrace’ process, which involves determining a single alignment from the extended anchor, as visually depicted in Figure 1C. The outcome of the extension (from Step 2) possesses multiple endpoints, from which one is selected by applying the criteria in the following order: the alignment (i) where either the query or target sequence is used to its end, (ii) that is longer and (iii) with the lesser penalty. After endpoint selection, operations are extracted sequentially from this endpoint to the anchor, the origin of the alignment. These operations fall into four categories—Match, Substitution, Insertion, and Deletion—collectively denoted as: Ω = {ω_*M*_, ω_*S*_, ω_*I*_, ω_*D*_}. Then, the side alignment for an anchor is expressed as a vector of operations:


\begin{eqnarray*} O_{n,\varphi } = \left[ o_0^{n,\varphi } , \ldots , o_{l_{\varphi }-1}^{n,\varphi } \right] \in \Omega ^{< \mathbb {N}}. \end{eqnarray*}

Therefore, the alignment, resulting from backtracing for *a*_*n*_, can be represented as:


\begin{eqnarray*} R_n = \left\langle a_n, O_{n,\varphi _L}, O_{n,\varphi _R} \right\rangle . \end{eqnarray*}

During backtracing, SigAlign identifies all anchors traversed by the alignment result. Let *T*_*n*_ denote the set of traversed anchors of anchor *a*_*n*_ and *T*_*n*, φ_ denote the set of traversed anchors on side φ. The notion that *a*_*m*_ traverses *a*_*n*_ implies that the side operation of *a*_*n*_ includes the position of *a*_*m*_, which can be represented as:


\begin{eqnarray*} \exists j \in \mathbb {N}_0: \; (i_{n,T} - i_{m,T})(i_{n,Q} - i_{m,Q}) > 0 \\ \wedge |i_{n,T} - i_{m,T}| = \sum _{i=0}^{j} [o_i^{n,\varphi } \ne \omega _D] \\ \wedge |i_{n,Q} - i_{m,Q}| = \sum _{i=0}^{j} [o_i^{n,\varphi } \ne \omega _I] \\ \Rightarrow a_m \in T_{n,\varphi } \end{eqnarray*}

(note: the square bracket is the Iverson bracket). Since SigAlign indexes the positions of anchors, each operation’s decision regarding traversed anchors is made in constant time *O*(1), as opposed to a linear time complexity of *O*(*n*), where *n* represents the number of anchors.

Subsequently, we deduplicate alignment results by assigning a ‘symbol’ to each result, as depicted in Figure 1D. SigAlign uses a hash value for the alignment’s position to enhance efficiency, rather than utilizing all crossed positions by *R*_*n*_. The symbol for alignment *R*_*n*_, denoted by ψ_*n*_, is derived by combining the starting anchor with its traversed side anchor sets:


\begin{eqnarray*} \psi _n = \left\lbrace a_n \right\rbrace \cup T_n. \end{eqnarray*}

SigAlign operates deterministically, meaning the alignment result between two anchors is always consistent, and the alignment extending from the last anchor (left-most or right-most by index in the sequence) is also identical. In other words, if the traversed anchors are the same, the alignment results will be the same. As such, the symbol acts as a unique identifier for the alignment result:


\begin{eqnarray*} \psi _n = \psi _m \Rightarrow R_n = R_m. \end{eqnarray*}

In addition, symbol ψ_*n*_ can also be interpreted as the set of anchors that become true-positive assuming *a*_*n*_ is included in true-positive. Thus, if *a*_*n*_ and *a*_*m*_ are mutually included in each other’s traversed anchors, the two propositions, ‘*a*_*n*_ is true-positive’ and ‘*a*_*m*_ is true-positive’, are identical, making the two anchors’ symbols also identical:


(8) \begin{equation*} \forall a_n, a_m \in A: \; (a_n \in T_m) \wedge (a_m \in T_n) \Rightarrow \psi _n = \psi _m. \end{equation*}

In summary, it can be affirmed that the alignment results of anchors mutually existing within each other’s traversed anchors are invariably identical, a characteristic exploited by SigAlign to swiftly deduplicate alignment results.

Lastly, using Equation (8) for result deduplication, we can predict the alignment results of anchors that haven’t been extended yet. Within SigAlign, the extension process is inherently bidirectional. Hence, even if *a*_*m*_ is found within the traversed anchors of *a*_*n*_, it does not necessarily imply that *a*_*n*_ is within the traversed anchors of *a*_*m*_ (∃*a*_*n*_, *a*_*m*_ ∈ *A*: (*a*_*n*_ ∈ *T*_*m*_)∧(*a*_*m*_∉*T*_*n*_)). However, even when the symbols of two anchors differ, if a common anchor is included in extensions performed in opposing directions by the two anchors, the symbol of the common anchor always matches the symbol of one of the two anchors. For instance, consider the scenario where two anchors, *a*_*c*_ and *a*_*m*_, are included in the right alignment of *a*_*n*_, and *a*_*m*_ is to the right of *a*_*c*_ (*i*_*c*, *Q*_ < *i*_*m*, *Q*_). In this case, since the extension progressing to the right from *a*_*n*_ has the same direction as the extension from *a*_*c*_, *a*_*m*_ is always included in the right alignment of *a*_*c*_ ($a_m \in T_{c,\varphi _R}$). Therefore, if the left alignment of *a*_*m*_ includes *a*_*c*_, then *a*_*c*_ and *a*_*m*_ have the same symbol. This can be represented as:


(9) \begin{equation*} \lbrace a_c, a_m\rbrace \subset T_{n,\varphi } \Rightarrow \forall a_c \in T_{m,\varphi ^\prime }: \; \psi _c = \psi _m. \end{equation*}

This implies that even if *R*_*n*_ and *R*_*m*_ are different, we can skip the extension of anchors lying within the intersection of ψ_*n*_ and ψ_*m*_.

Efficiently skipping redundant anchors using Equation 9 requires prioritizing anchors within an evaluated anchor’s alignment result for extension. This process, however, can unfold recursively, adding complexity. Tracking all anchors recursively would necessitate additional calculations and memory space for recording the order of anchor extensions. Therefore, a specific procedure is adopted to manage the order of anchor extensions. This approach ensures the extension and evaluation of anchors proceed from left to right and limits the recursive modification of anchor extension order to a single occurrence. For a detailed description for anchor processing order, refer to Supplementary Note 7.

### Performance evaluation

To comprehensively assess the performance of SigAlign across various dimensions, we organized our tests as follows (each number corresponds to the respective subsections in the ‘Results’ section):

1. Read mapping across various sequencing data: We assessed the performance of SigAlign using twofold tests: first, by mapping actual sequencing reads obtained from multiple sequencing platforms to evaluate performance variations across different data types. Second, we examined the accuracy of these aligners by mapping simulated reads, which were generated based on the reference genomes of a diverse set of organisms.
2. Application in database search: To evaluate performance on a database of an integrated reference of microbes, we assessed the accuracy of measuring relative abundance of taxa. Relative abundance was calculated by mapping simulated metagenome sequences with known abundance to assess the accuracy. To further evaluate the performance in database search, an additional test for the ‘dilution’ of search results was conducted. This tested whether alignment results for a single genome are not diluted at the presence of multiple similar genomes in database, thereby evaluating the usefulness in the real-world tasks involving large databases with similar sequences.
3. Memory footprint: We quantified SigAlign’s memory usage under various conditions—number of reference chunks, threads, and query lengths—to evaluate resource efficiency and the size of the workspace per thread (methodology for memory profiling is detailed in Supplementary Note 8).

#### Aligners for comparative performance tests

Alongside SigAlign, we evaluated a range of widely used heuristic aligners. The tools in comparison include four primarily used for read mapping—BWA-MEM (6), bowtie2 (7), HISAT2 (30) and minimap2 (31)—and two mainly used for database search—BLASTn (8) and MMseqs2 (32). A broader comparison with other aligners is discussed in the ‘Discussion’ section.

Tools were applied with their ‘default’ options, albeit with a few exceptions. Firstly, to ensure a balanced comparison, we specified the use of a single thread if the default options included multi-threading. Secondly, exclusively for recall tests in large databases (’Recall the alignment results in large database’ subsection), we applied an additional option to output all alignment positions for tools other than SigAlign. Versions and commands for all aligners, excluding SigAlign, are detailed in Supplementary Table S2.

#### Default configurations for SigAlign

In this comparative test, we developed and utilized a command-line interface (CLI) demonstration binary of SigAlign to ensure compatibility with other aligners, although SigAlign is originally developed as an open-source library allowing an integration to other tools. Detailed information on the specific version and compilation method of this binary is provided in Supplementary Note 9, with the commands used for testing detailed in Supplementary Table S3.

Instead of setting SigAlign’s five parameters differently for each test, we designated two representative parameter sets: *strict* and *lenient*. The *strict* set, compared to the *lenient* set, employs a higher MinL and a lower MaxP, expecting faster speeds but yielding fewer results due to only outputting alignments of higher similarity. To maintain stable throughput, we used the square root of the query sequence length as a scaling factor for the cutoff, considering the algorithm’s workload is roughly proportional to the square of the query sequence length. Specifically, for a query length *l* (in base pairs), the *strict* set uses $5\sqrt{l}$ and $0.5/\sqrt{l}$ for MinL and MaxP, respectively, while the *lenient* set uses $2\sqrt{l}$ and $1.5/\sqrt{l}$. The gap-affine penalty for both parameter sets equally applies mismatch, gap-open, and gap-extend penalties of 4, 6 and 2, respectively, set within a range similar to other aligners (Supplementary Table S4).

#### Reference and query sequences

For read mapping tasks, we utilized both real and simulated genome datasets to ensure validation through simulated data’s validity metrics. In the task involving actual sequencing reads, *Mycobacterium tuberculosis* (*M. tb*) H37Rv (33) and *Homo sapiens* T2T-CHM13 (34) were used as reference genomes. Short-read queries for *M. tb* were derived from Illumina’s NovaSeq (PRJEB49562) and MiSeq (PRJEB35201), while long-read queries for the human genome were obtained from Oxford Nanopore Technologies’ (ONT) MinION (R10.4 flowcell, PRJNA875576) and Pacific Biosciences’ (PacBio) Sequel II (HiFi reads, PRJNA529679). Query sequences were extracted at regular intervals from each project, restricted to a range that encompasses average read lengths to prefilter outliers (the extraction process and statistics of original reads are described in Supplementary Note 10). For simulated read mapping, reference genomes for nine species were sourced from the NCBI RefSeq database, specifically: *Mycobacterium tuberculosis* (GCF_000195955.2), *Escherichia coli* (GCF_000008865.2), *Saccharomyces cerevisiae* (GCF_000146045.2), Arabidopsis thaliana (GCF_000001735.4), Drosophila melanogaster (GCF_000001215.4), *Oryza sativa* (GCF_001433935.1), *Danio rerio* (GCF_000002035.6), *Mus musculus* (GCF_000001635.27) and *Homo sapiens* (GCF_009914755.1). Each query was simulated from its corresponding reference using Mason’s Illumina short-read profile (35).

The database search test employed the Unified Human Gastrointestinal Genome (UHGG) (36) database. For abundance profiling, references comprised the top 200 and 100 most prevalent genera in the human gut, based on the ‘prevalence score’ from the HumGut (37) database. Queries comprised 50 million reads, with 200 most prevalent genera contributing an equal proportion, simulated using InSilicoSeq (38). The average per-base accuracy of the simulated reads, measured by Phred score, was 98.45%. For recall tasks within a large database, references were constructed with 4096 randomly selected *Escherichia coli* genomes from the UHGG database. The number of genomes included doubled from 1 to 4096 in each subsequent reference, ensuring that larger references encompassed all strains present in their smaller counterparts. For queries, we extracted every 300 bp fragment from a single *E. coli* genome not included in the references, starting from the first base pair and proceeding with a stride of 11 bp, yielding 485 353 fragments. The exact genome IDs for these references and the query are listed in Supplementary Note 11.

For the memory footprint test, we utilized a reduced representative genome set from the UHGG database as the reference. This set is large enough to test the algorithm’s performance effectively while not representing a specific genome. We combined genomes from each taxonomic ‘class’ within the UHGG database, resulting in a dataset of 84 genomes comprising 11 709 contigs with a total size of 235 Mbps. Supplementary Note 11 provides the exact genome IDs for this reference. To assess performance across various query lengths, we simulated sequences from this reference using dwgsim (https://github.com/nh13/DWGSIM), with each simulation producing 1 000 000 reads.

Supplementary Table S5 details the query sequences, encompassing read counts and descriptive statistics. Supplementary Table S6 delves into the reference sequences, with a particular emphasis on the building (or indexing) time. For additional information, the versions and commands used for query simulation are documented in Supplementary Note 12.

#### Machine specifications and constraints

The testing was conducted on a system with the following specifications: (i) OS—Ubuntu 20.04; (ii) CPU—Intel(R) Xeon(R) CPU E5-2680 v4 (2.4 GHz, 34 MB) * 2; (iii) RAM—DDR4 2133 MHz (total: 32 GB * 8 = 256 GB); (iv) Storage—SATA3 (6 Gbps)/RAID 5.

We applied several constraints to curtail test bias. To minimize the impact of file loading and program initialization on the measured speed, we combined the queries into a single FASTA file. For the reference sequences, we used ‘pre-built’ sequences that underwent a preparation phase, as they can be reused multiple times, thus having a minimal effect on the overall speed. To avoid performance fluctuations due to system overload, we ensured that neither the system core and memory were fully utilized, nor did the I/O speed exceed half of its maximum capacity throughout the test. We logged the elapsed time to execute a command, doing so a minimum of 15 times for tasks taking under 1000 seconds and 5 times for those taking up to 3600 s. Throughput was calculated using the median value of elapsed time.

## Results

### Read mapping across various sequencing data

#### Performance for diverse sequencing platforms

Comparative evaluations in our research focused on throughput (reads processed per second), identity (the percentage of matching base pairs compared to the total length of alignment), and mapping rate (the proportion of reads mapped from the total reads submitted).

Table 1 summarizes the findings, highlighting a more pronounced performance disparity in long-read alignments compared to short reads. For short reads, all tools showed high identity (>98%, except MMseqs2 on MiSeq) and mapping rates (>90%, except for HISAT2 on MiSeq). However, for long reads, both identity and mapping rates varied widely between tools. Throughput disparities also widened significantly, from a 50-fold gap between the slowest and fastest tools for short reads to over 50 000-fold for long reads. This variation allows for the classification of tools into three groups based on their performance shift from short to long reads.

**Table 1.** Read mapping performance across sequencing platforms

	Group 3			Group 2*			Group 1	
	SigAlign (*strict*)	SigAlign (*lenient*)	HISAT2	BLASTn	MMseqs2	Bowtie2	BWA-MEM	Minimap2
***Mycobacterium tuberculosis*(H37Rv)**								
**Illumina NovaSeq (90–101 bp, 99.86%)**								
**Throughput (read/s)**	193 387	2914	77 194	6514	4630	11 101	29 893	39 743
**Identity (%)**	99.89	99.53	99.89	99.62	98.01	99.86	99.85	99.83
**Mapping rate (%)**	91.97	92.96	90.05	91.94	92.76	92.80	92.81	92.50
**Illumina MiSeq (250–301 bp, 98.79%)**								
**Throughput (read/s)**	92 338	54 012	24 868	4621	1945	1026	7564	9836
**Identity (%)**	99.72	99.23	99.44	98.33	95.60	99.01	99.05	98.86
**Mapping rate (%)**	96.63	99.58	83.75	99.52	99.70	99.61	99.63	99.58
***Homo sapiens*(T2T-CHM13)**								
**ONT MinION (3000–4000 bp, 95.59%)**								
**Throughput (read/s)**	1765	43.34	90.78	0.0277	0.0184	0.3649	32.44	118
**Identity (%)**	99.90	99.55	99.13	82.51	80.03	73.25	97.30	95.21
**Mapping rate (%)**	74.88	88.51	5.13	98.70	99.65	98.05	97.98	98.01
**PacBio Sequel II (12 000–14 000 bp, 99.81%)**								
**Throughput (read/s)**	450	11.10	9.54	0.0068	0.0071	0.0094	3.91	22.98
**Identity (%)**	99.95	99.77	99.78	81.83	80.22	74.90	99.41	98.17
**Mapping rate (%)**	93.95	99.78	43.74	99.70	99.90	99.90	99.89	99.88

Performance metrics—throughput, identity, and mapping rate—were evaluated for short reads from Illumina NovaSeq and MiSeq platforms mapped to the bacterial genome (*Mycobacterium tuberculosis*, H37Rv), and for long reads from ONT MinION and PacBio Sequel II platforms mapped to the human genome (*Homo sapiens*, T2T-CHM13). Read lengths and base call accuracy, as inferred from Phred scores, are noted in parentheses alongside each sequencing platform. Alignment tools are categorized into three groups based on their performance with human long reads: stable (Group 1); detecting lower similarity with reduced throughput (Group 2); and fast with lower mapping rates (Group 3). Identity and mapping rate values are displayed to two decimal places. Throughput is rounded to four decimal places for values under 1, has no decimal places for values over 100, and to two decimal places for values in between.

*For human long reads, tools in Group 2 used 1/100 sampled reads due to limited throughput.

The first group includes BWA-MEM and minimap2, demonstrating stable and moderately high performance across all metrics for long reads, with identity >95% and mapping rates >97%, while maintaining reasonable processing speeds (minimap2 as the second fastest and BWA-MEM as the fifth fastest among nine tools).

The second group, consisting of BLASTn, MMseqs2, and bowtie2, achieved the highest mapping rates (>98%) and the lowest identities (<83%), facing a steep decline in throughput (<0.5 reads/s). These tools were slower for short reads, and this gap widened for long reads.

The third group, SigAlign and HISAT2, exhibited the fastest speeds, with the highest identity (>99%) but a drop in mapping rates. HISAT2’s mapping rate decreased across both ONT MinION (∼5%) and PacBio Sequel II (∼44%). SigAlign, in contrast, showed a mapping rate decrease mainly in the ONT MinION dataset (74–89%), which is less accurate, while maintaining relatively high mapping rates (>93%) for the more accurate PacBio Sequel II data, indicating sequencer accuracy impacts mapping rates more than read length for SigAlign.

SigAlign’s performance showed significant variation between *strict* and *lenient* parameter sets. The *strict* achieved the highest throughput, more than doubling HISAT2’s for short reads and exceeding minimap2’s by over ten times for long reads. In contrast, the *lenient* led to higher mapping rates and alignments with lower similarity.

#### Accuracy in simulated short reads across organisms

To evaluate alignment accuracy, we simulated 300 bp reads from the genomes of nine organisms: *M. tuberculosis*, *E. coli*, Yeast, Thale Cress, Fruit Fly, Rice, Zebrafish, Mouse and Human. These reads were subsequently aligned back to their original reference genomes. Given that a single read can yield multiple alignment outcomes, we assessed accuracy from two comprehensive perspectives: sensitivity and precision.

Sensitivity, considered from the read’s perspective, is the proportion of reads for which at least one alignment correctly matches the original simulated position. This measure is calculated by dividing the number of correctly aligned reads (true positives) by the total number of reads (positives). Precision, evaluated from the alignment’s perspective, assesses whether each proposed alignment accurately corresponds to its intended position. This is quantified by dividing the number of correct alignments (true positives) by the total number of alignments proposed (predicted positives). Correctness of alignments is determined by whether their positions overlap with the simulated positions, with detailed method provided in Supplementary Note 13.

**Figure 2.**
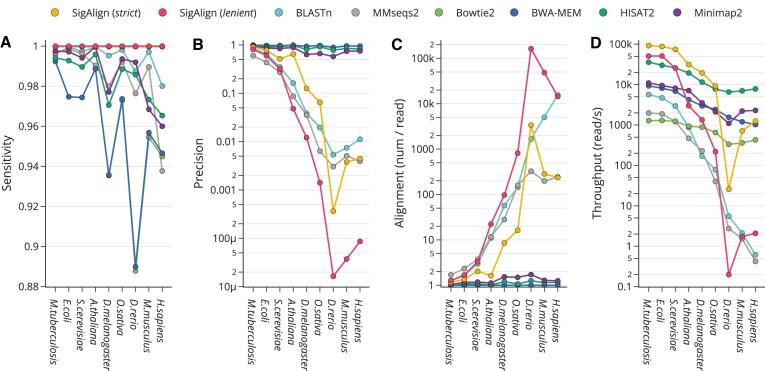
Performance of alignment algorithms on simulated 300 bp reads across nine genomes, with the organisms ordered by genome size (on the x-axis). Sensitivity (**A**) reflects the proportion of reads that are correctly aligned to at least one position that matches the simulated origin. Precision (**B**) is the proportion of correct alignments out of all proposed alignments. (**C**) shows the average number of alignments per read. (**D**) depicts the throughput, measured as the number of reads processed per second. For (B)–(D), the y-axis is on a logarithmic scale. For the three largest genomes—*D. rerio*, *M. musculus*, *H. sapiens*—performance data for SigAlign (*lenient*), BLASTn and MMseqs2 are based on 1/100 of sampled reads due to restricted throughput.

Figure 2 presents the results, including sensitivity (Figure 2A), precision (Figure 2B), the average number of alignments per read (Figure 2C), and throughput (Figure 2D), sorted by the total length of the reference genomes. Regarding sensitivity, most tools exhibited high performance (>0.88), as shown in Figure 2A, with SigAlign achieving the highest sensitivity across all organisms, notably reaching perfect sensitivity (1.0) with *lenient* cutoff. However, precision showed substantial variation among the tools (Figure 2B). Read mappers—BWA-MEM, bowtie2, HISAT2, and minimap2—consistently delivered high precision (>0.55), unaffected by genome size. In contrast, tools designed primarily for database searches—BLASTn, MMseqs2—and SigAlign experienced a notable decline in precision as genome size increased. This difference in precision stems from operational distinctions: read mappers prioritize finding the most accurate single alignment per query, resulting in fewer than 1.8 alignments per read, whereas database search tools and SigAlign strive to identify all potential match locations (Figure 2C). Throughput analysis revealed that read mappers (BWA-MEM, bowtie2, HISAT2 and minimap2) exhibited more consistent performance compared to other tools (Figure 2D).

In summary, SigAlign, through its non-heuristic approach, outputs all alignments that surpass the specified cutoffs (MinL and MaxP). This leads to a decrease in both precision and speed as the number of potential alignments increases, similar to what is observed with database search tools. Consequently, SigAlign (*strict*) maintained the highest throughput up to the Rice genome (*O. sativa*) but lost this advantage with larger genomes. However, SigAlign consistently showed the highest sensitivity across all genomes evaluated. Using a *strict* cutoff results in lower sensitivity compared to *lenient*, but the consistency in sensitivity is preserved irrespective of the cutoff used. This consistent sensitivity level was also observed under various cutoff settings beyond the *strict* and *lenient*, as detailed in Supplementary Table S7.

### Application in database search

#### Human gut metagenome abundance profiling

**Figure 3.**
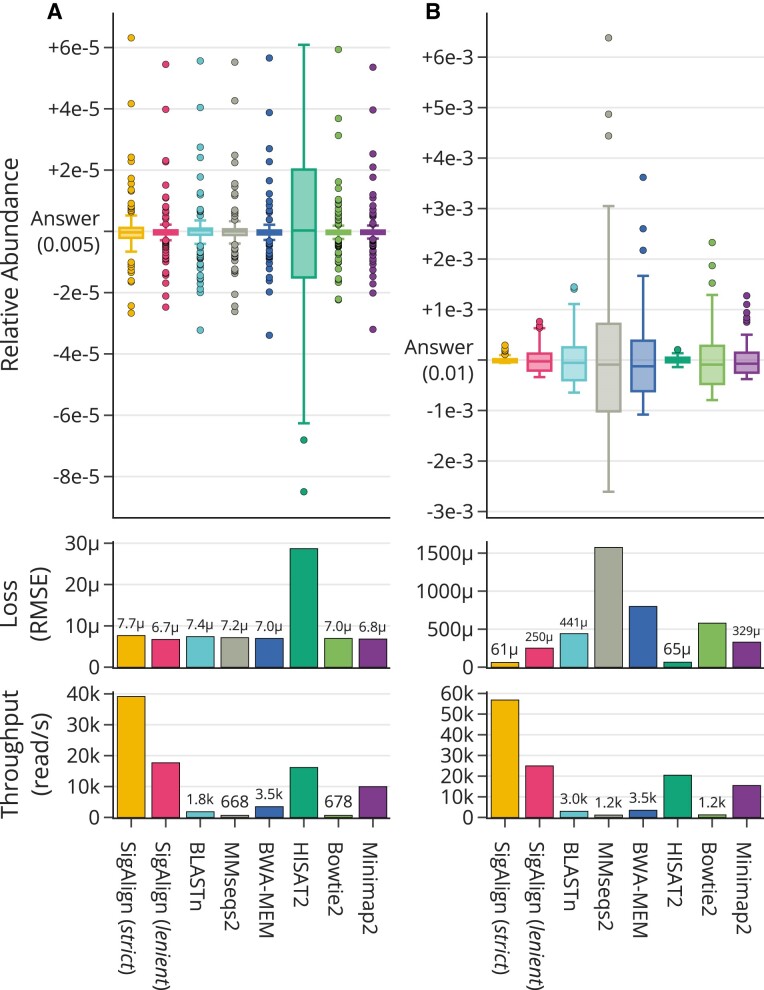
Comparative performance of alignment algorithms in human gut metagenome abundance profiling using two databases: (**A**) a full database containing the top 200 genera from which queries were simulated, and (**B**) a restricted database comprising the 100 most prevalent genera. The top panels display the relative abundance distributions, with all data points contained within the plot bounds and the ideal correct ratio marked as ‘Answer’ for deviation comparison. The middle panels present the loss, quantified by RMSE (root mean square error), based on the ‘Answer.’ The bottom panels show throughput, represented as the number of reads processed per second.

We generated a query set by simulating an equal number of sequences from each of the 200 most prevalent genera in the human gut. These queries were aligned against a ‘full database’, i.e. the original data source (Figure 3A), and a ‘restricted database’ containing only the top 100 most prevalent genera, simulating some genera present in the sample but not in the database (Figure 3B). The relative abundance was determined by the ratio of queries mapped to each genome, with the calculation methods detailed in Supplementary Note 14. We also tested a database comprising the top 400 genera, and these results are provided in Supplementary Figure S3.

We evaluated the accuracy of the alignment tools using the Root Mean Square Error (RMSE), a commonly used metric for quantifying the loss or error in predictive models. RMSE is calculated as follows: $\text{RMSE} = \sqrt{\frac{1}{n} \sum _{i=1}^{n} (\text{predicted}_i - \text{observed}_i)^2}$, where *n* is the number of genomes present in the database, predicted_*i*_ is the predicted relative abundance, and observed_*i*_ is the actual observed abundance for the *i*th genome. Lower RMSE values indicate higher accuracy. The exact RMSE values, along with another loss metric, Mean Absolute Error (MAE), are provided in Supplementary Table S8.

In the full database, all tools except HISAT2, which showed the lowest accuracy (RMSE > 28μ), exhibited similar accuracy levels (RMSE around 7μ ± 1μ). However, in the restricted database, the differences in accuracy between the tools were pronounced (Figure 3B). In the restricted database, SigAlign (*strict*) and HISAT2 demonstrated the highest accuracy, with RMSE values less than 70μ, which was more than three times lower than other tools. Following these, SigAlign (*lenient*), minimap2, BLASTn, and BWA-MEM showed progressively lower accuracy, while MMseqs2 had the lowest accuracy with RMSE values at least twice as high as the other tools. Notably, excluding HISAT2, SigAlign had the smallest increase in RMSE when transitioning from the full database to the restricted database, both in terms of absolute increase and relative factor.

Regarding speed, most tools performed faster on the restricted database compared to the full database, except for BWA-MEM, which showed a slight slowdown (about 1.4%) in the restricted database. SigAlign (*strict*) was the fastest, achieving at least twice the processing speed of other tools. Following this, SigAlign (*lenient*), HISAT2, and minimap2 were the next fastest. The remaining tools showed reduced speed, being more than twice as slow as minimap2.

#### Recall of alignment results in large database

SigAlign’s non-heuristic approach ensures that alignment results for any reference genome are always included in the output from databases that contains that genome. This capability is particularly advantageous when dealing with large databases hosting a vast array of similar genomes, such as the NCBI ‘Genome’ database, which comprises approximately 250 000 *E. coli* genomes. The primary challenge in such extensive databases is the potential for specific genome alignment results to be missed by the sheer number of similar sequences.

To simulate this challenge, we constructed reference databases with *E. coli* strains ranging from 1 to 4096, with each larger database encompassing all strains from the smaller ones. A query set of short (300 bp) reads, extracted from an *E. coli* strain not present in any reference database, was aligned against each database. We measured the recall rate of alignment results for each database against two standards: (i) the initial single-strain reference, and (ii) the next smallest reference database, with half the number of strains (true positive: correctly identified; false negative: overlooked). Comparative analysis was conducted with commonly used database search tools like BLASTn, MMseqs2 and bowtie2, configured to report all possible alignments. Configuration details are in Supplementary Table S2, with additional results from other aligners in Supplementary Figure S4.

**Figure 4.**
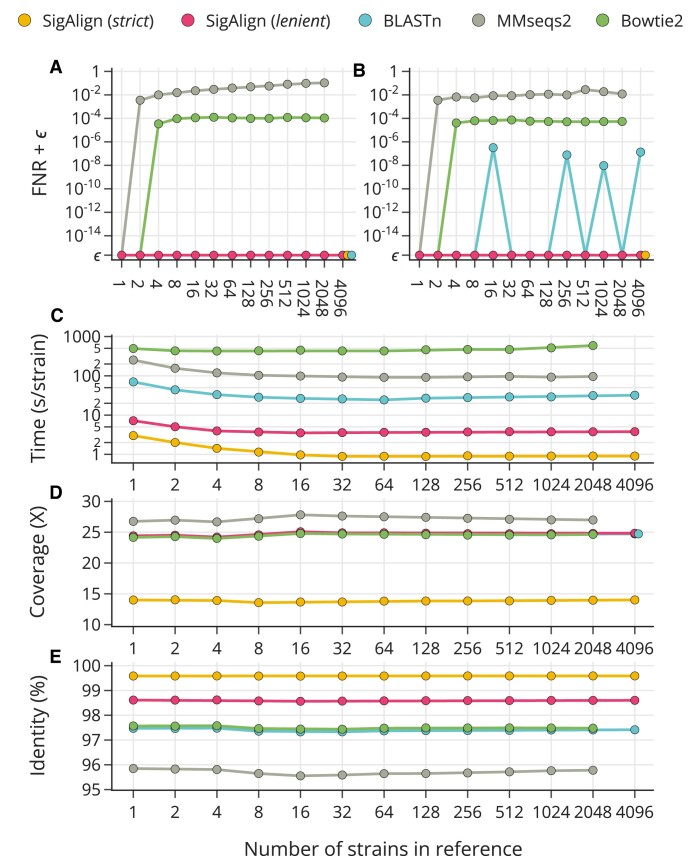
Recall performance in large database. (**A**) and (**B**) display the False Negative Rate (FNR) for the smallest reference containing a single strain (A) and for a half-sized reference with half the strains (B). The FNR is incremented by ε (the machine epsilon for double-precision floating-point numbers, 2.22045e-16) and presented on a logarithmic scale to clearly delineate performances, including the zero value. (**C**) shows the processing time (in seconds) per strain, on a logarithmic scale. (**D**) displays coverage, defined as the total output length divided by the reference length. (**E**) presents percent identity, calculated as the total matched base pairs divided by the total output length. The x-axes represent the number of strains in the reference on a logarithmic scale.

The false negative rate (FNR) for alignments against the initial and half-sized reference databases is detailed in Figure 4A and B, respectively, with exact values available in Supplementary Table S9. SigAlign achieved a perfect recall rate (FNR = 0) under both *strict* and *lenient* cutoffs for both scenarios. BLASTn maintained an FNR of 0 for the initial reference but showed slight increases in FNR (from 10^−6^ to 10^−8^) across the four half-sized databases (details of BLASTn’s missed alignments are in Supplementary Note 15). Following BLASTn, bowtie2’s recall rate was approximately 100 times higher than that of MMseqs2, with bowtie2 and MMseqs2 consistently showing FNRs of 10^−4^ and 10^−2^, respectively.

Additional metrics evaluated included speed, coverage and identity. Speed was measured as the alignment time per strain in the reference database, with SigAlign outperforming competitors by at least 20 times in *strict* settings and over six times in *lenient* settings (Figure 4C). A trade-off was observed between coverage—defined as the total alignment length compared to the reference genome size (Figure 4D)—and identity, which is the percentage of matched base pairs within the total alignment length (Figure 4E). SigAlign (*strict*) showed the highest identity and lowest coverage, while MMseqs2 exhibited the highest coverage but the lowest identity. SigAlign (*lenient*), BLASTn, and bowtie2 displayed similar coverage values, with SigAlign (*lenient*) achieving slightly higher identity (about 1%).

### Memory footprint

#### Memory usage by the number of reference chunks

Typically, when the size of a reference is large, it is divided into several chunks for processing. We measured the memory usage of SigAlign by varying the number of reference chunks (from 1 to 32). For the reference, we used a reduced combined genome consisting of one genome per taxonomic class from the UHGG database, and for the query, we used simulated 1000 bp sequences.

**Figure 5.**
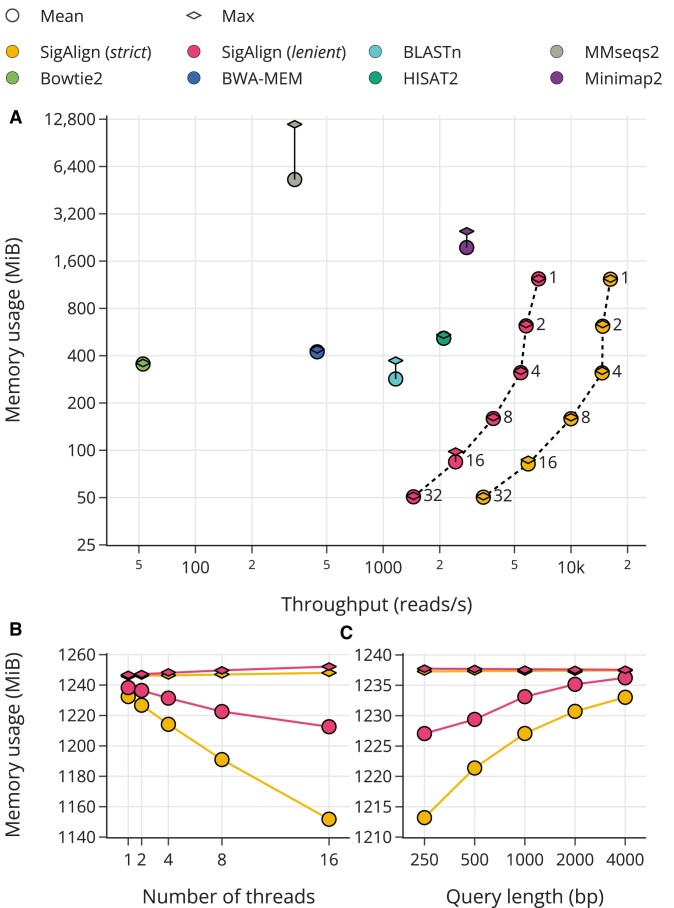
Memory footprint. The color of the marker identifies the tool, while the shape indicates whether the value represents mean (circle) or maximum (diamond) memory usage. (**A**) illustrates memory usage in relation to the number of reference chunks in SigAlign, ranging from 1 to 32, with the specific number of chunks labeled next to the corresponding marker. (**B**) and (**C**) demonstrate the memory usage of SigAlign relative to the number of processing threads (B), and query length (C). Default settings for measurements unless specified: query length at 1000 bp, single reference chunk, and one processing thread.

SigAlign demonstrated improved memory efficiency under test conditions, showing both faster speed at comparable memory usage and lower memory usage at equivalent speeds (Figure 5A). Additionally, it was observed that for SigAlign, the benefits of reduced memory usage outweighed the drawbacks of reduced speed when using a small number of chunks (from 1 to 8 chunks). Notable case in this test is that, when increasing the number of chunks from 2 to 4 in the *strict* set, the average memory usage decreased by 49.56%, while the speed decreased by only 0.65%. Moreover, SigAlign was shown to operate in small memory space about 50 MiB, when using the 32 chunks. The time series memory usage for all tools is presented in Supplementary Figure S5.

#### Runtime workspace size per thread

We calculated the size of the runtime workspace for SigAlign—the memory space used for actual alignment tasks beyond the space occupied by reference loading. Since the reference in SigAlign can be shared among threads, the size of the workspace meant that the memory usage increase for each threads in multi-thread environment. Although each thread can operate with different parameter sets (e.g. one of two multi-threaded operations might use the *strict* parameter set while the other uses the *lenient* set), we used same parameters set for all threads for simplification for the test.

We measured the memory usage based on the number of threads using the same reference and 1000 bp query as in the previous subsection (Memory usage by the number of reference chunks) (Figure 5B). When assuming that the maximum memory usage increases linearly with the number of threads, the workspace size per thread estimated by maximum memory usage is 154.1 KiB and 369.6 KiB for the *strict* and *lenient* parameter sets, respectively. This suggests that a 10 MiB memory space can support around 66 and 27 threads simultaneously for the *strict* and *lenient* parameter sets, respectively. There also shown a decrease in mean memory usage by thread count which is attributed to the increased speed by multi-processing (detailed in Supplementary Table S10).

We also assessed that how does this workspace size be effected by the length of the query. In alignments using simulated queries ranging from 250 to 4000 bp on the same reference, the maximum memory usage fluctuated within 0.4 MiB (Figure 5C). This memory usage variation about length of the query of SigAlign is shown to be minimal compared to other tools (Supplementary Figure S6).

## Discussion

### Advantages of non-heuristic results

SigAlign’s innovation lies in its departure from the traditional reliance on technical, algorithm-specific heuristics to manage throughput. Instead, SigAlign adopts two straightforward cutoffs: the minimum alignment length (MinL) and the maximum penalty per alignment length (MaxP), along with three affine gap penalties. Such a design not only facilitates control over throughput but also makes SigAlign’s process inherently non-heuristic.

SigAlign’s non-heuristic approach facilitates straightforward adjustments and predictions regarding alignment results. Its parameters are not only biologically relevant but also distinctly outlined, ensuring outcomes are predictable and modifications are guided clearly. For instance, if certain parameters yield no alignment results, users can be confident that applying stricter cutoffs (higher MinL or lower MaxP) will also not produce results. On the other hand, if the output is abundant, employing stricter cutoffs is likely to improve both precision and processing speed. Unlike systems where parameter effects are ambiguous, SigAlign’s five parameters are transparent, reducing the need for empirical parameter space exploration. Additionally, SigAlign guarantees consistent alignment outcomes regardless of target sequences’ locations in the reference, offering a robust solution that eliminates redundant efforts (Figure 4A, B, Supplementary Figure S4). These features make SigAlign advantageous for adapting to new research domains by flexibly and quickly adjusting its results and performance.

Furthermore, SigAlign’s non-heuristic approach simplifies the reproducibility and communication of results. The results from SigAlign are comparable to filtering the alignment results of traditional non-heuristic aligners through specific cutoffs (see Supplementary Note 3 for more details). Consequently, SigAlign’s results remain stable across software updates, as long as the definition itself does not change. This stability allows SigAlign to describe the alignment results in clear, algorithm-agnostic terms. For example, the results obtained with SigAlign’s parameters (MinL: 200; MaxP: 0.08, when a mismatch penalty is 4) can be seamlessly expressed as ‘alignments of at least 200 bp in length with up to two mismatches (or equivalent gaps) per 100 bp’. This clarity not only enables subsequent analyses and the development of new tools to rely on a more transparent alignment process but also lowers the barrier for professionals outside of sequence alignment to engage and contribute their insights.

### Appropriate tasks for SigAlign

Comprehensive testing has illuminated several tasks where SigAlign’s design and functionality significantly enhance performance. One such task is the rapid and accurate identification of alignments with high similarity. In cases where a sequencer’s error rate is low and high similarity is generally expected, SigAlign’s ability to apply a stricter cutoff (*strict* in our tests) resulted in the fastest speed for both short and long reads (<14 kb) (Table 1). Presently, high-accuracy sequencers are predominantly utilized, a context in which the advantages of SigAlign become particularly evident. SigAlign’s throughput is especially high when there are only a few expected locations for a query to map in the reference genome, such as in bacterial genomes with relatively scarce repeat regions. SigAlign consistently showed the fastest speeds in all these cases (Table 1, Figures 2D, 3, 4C and 5A).

Moreover, SigAlign is highly suitable for tasks involving classifying queries into their exact positions. It can accurately locate the original position from which a read is derived, regardless of the reference genome (Figure 2A). By adjusting cutoffs, SigAlign has the capability to leave queries unaligned when they should not map to a reference (Figure 3A). This feature can be useful in several instances. For example, in read mapping tasks, such a capability can be used for removing contaminant reads or handling reads dissimilar to the reference via de-novo assembly. Additionally, this is beneficial in metagenomics, where samples often contain taxa or genes not represented in the reference genome.

SigAlign is particularly suited for high scalability tasks. It supports segmenting a vast reference genome into smaller pieces for alignment, ensuring consistent results when these segments are later merged (Figure 4A, B). For instance, SigAlign can conduct targeted alignments for only a portion of the reference, such as the X chromosome of the human genome or specific coding sequences, achieving results equivalent to those from aligning with the full reference. Given the trend towards horizontal scalability in contemporary computing systems, SigAlign’s compatibility with distributed tasks —free from detrimental side effects —underscores its utility in such environments. Moreover, SigAlign’s small and adjustable memory footprint contributes to its suitability in distributed systems (Figure 5A). From a software engineering perspective, SigAlign is optimized for multi-threaded operations. Since the reference object in SigAlign is immutable (unchanged during runtime), it is safe for multiple threads to access, avoiding race conditions. Additionally, SigAlign can simultaneously run >20 additional aligners in about 10MiB of space (Figure 5B, C). Thus, SigAlign is well-suited for scaling tasks from both system and thread perspectives.

### Other aligners beyond the test settings

In our tests, we focused on comparing heuristic aligners that utilize the affine gap penalty scheme. However, various heuristic aligners employ a wide range of scoring schemes, from the simpler edit (or Levenshtein) distance, to more complex substitution matrices, which apply different scores for specific protein substitutions (39) or DNA purine/pyrimidine changes (40,41). Although complex scoring schemes can reflect more complex biological phenomena, they require more computational resources, impacting the alignment speed. Within the context of DNA alignment, the affine gap penalty system is commonly used for its ability to balance biological relevance—by encouraging consecutive gaps—with computational efficiency. SigAlign and the other tools discussed in this paper utilize this gap-affine penalty scheme.

Among existing aligners, RazerS (28) is conceptually similar to SigAlign, employing a clear cutoff based on sequence identity. However, unlike most aligners that support ‘local’ alignments, which allow for the use of sequence substrings, RazerS supports only ‘end-to-end’ (or semi-global) alignments. Furthermore, RazerS does not construct an index of the reference genome and relies solely on the Hamming and edit distances for its scoring schemes, not on affine gap penalties. Consequently, RazerS was excluded from our comparative tests due to its significantly different alignment outcomes compared to other aligners. Results from RazerS’s tests on the read mapping dataset are available in Supplementary Table S11.

### Future directions

The primary goal of SigAlign is to enhance the development of bioinformatics tools and pipelines by serving as a key internal component. Our immediate focus is on evolving SigAlign into a comprehensive open-source library that not only delivers high performance but also features a well-structured interface and user-friendly functionalities. SigAlign already supports several functions designed to improve flexibility and usability, including compatibility with different sequence storage formats, the ability to modify search ranges during runtime, and the capability to compress and distribute the reference object. As users of SigAlign ourselves, we plan to develop analysis pipelines that incorporate SigAlign as a module, allowing us to continuously update it and directly contribute to the biological knowledge base.

### Limitations

We admit that SigAlign presently has several limitations. First, the mapping rate diminishes for the low-accuracy reads, such as those from MinION platforms (Table 1). Adjusting the parameters as *lenient*—decreasing MinL and increasing MaxP—can improve the mapping rate, but at the cost of decreased throughput.

Second, SigAlign’s throughput drops when encountering numerous alignments that meet the predefined cutoffs (Figure 2C-D). This pattern is observed with other alignment tools as well, but SigAlign is more vulnerable to this challenge, particularly under the *lenient* parameter setting. For instance, the drops in throughput were evident when dealing with queries from repetitive sequences, or references with numerous duplicates.

Third, SigAlign’s precision decreases when the number of alignments per query is large (Figure 2B, C). However, SigAlign can improve precision by selecting one ‘primary alignment’ for each query, similar to read mappers. For example, selecting the alignment covering the longest query length (and in cases of equal query lengths, the one with the smaller penalty) as the ‘primary alignment’ for each query allows SigAlign to achieve higher precision in all genomes, except under the *strict* parameter setting on the FruitFly genome. Supplementary Table S12 provides the detailed values for precision its and calculation methods.

Fourth, SigAlign has proven effective at aligning sequences within the 12–14 kb range, particularly with higher-accuracy reads (evidenced by PacBio’s HiFi sequences in Table 1). However, SigAlign’s speed decreases with longer query sequences, making queries larger than this range challenging. For instance, ONT may produce reads exceeding 1 Mb; tools optimized for very long reads (e.g. minimap2) rather than SigAlign are recommended. Additionally, for comparing large genomes, SigAlign may need to be supplemented by the use of ANI (average nucleotide identity) tools, which involve dividing the genome into multiple smaller fragments for alignment (42).

Finally, SigAlign is currently suited for DNA sequencing data and is not optimized for RNA or amino acid data. To accommodate these, implementations such as RNA splicing indicators or a substitution matrix for amino acids—considering their chemical properties—are necessary for future development.

### Conclusion

SigAlign is a novel algorithm that employs straightforward, biologically relevant cutoffs to deliver non-heuristic results with the capability of speed regulation. It bridges the gap between the transparency of non-heuristic aligners and the efficiency of heuristic aligners, providing clear and understandable outcomes without compromising on performance. Extensive testing has established SigAlign’s niche in the realm of biological sequence analysis, an area traditionally dominated by heuristic methods. In particular, SigAlign excels in identifying high-similarity alignments rapidly and accurately, especially when dealing with data from low-error-rate sequencers such as Illumina or PacBio HiFi. It is also well-suited for tasks involving the classification of query sequences into their exact positions, such as in metagenomics analysis. We anticipate that SigAlign will serve as a catalyst for the development of new tools and analytical methods, offering a balanced solution that respects the integrity of biological data while accommodating the practical need for efficiency.

## Supplementary Material

gkae607_Supplemental_File

## Data Availability

SigAlign is a publicly accessible open-source library, disseminated under the provisions of the MIT license, a permissive license that promotes free software usage. The library is implemented in the Rust programming language. The source code of SigAlign referenced in this paper can be accessed on Zenodo (https://doi.org/10.5281/zenodo.10253841). The complete source code, accompanied by a detailed manual and examples for other languages such as Python or JavaScript, is available on the GitHub repository (https://github.com/baku4/sigalign/). Comprehensive API documentation detailing its functionalities can be found via the Rust library registry (https://docs.rs/sigalign/). For full details on the dataset and software specifications employed in our analysis, please refer to the ‘Materials and Methods’ section.
