# Supplementary material for: SigAlign: an alignment algorithm guided by explicit similarity criteria
Source: Nucleic Acids Res. 2024 Jul 16;52(15):8717–33. doi: 10.1093/nar/gkae607 (PMC11347165; doi:10.1093/nar/gkae607)
Supplement: gkae607_Supplemental_File [file gkae607_supplemental_file.pdf]

<u>Supplementary Information</u>

SigAlign: An Alignment Algorithm Guided by Explicit Similarity Criteria

Kunhyung Bahk

Last Updated: 2024-06-15

Table of Contents

Supplementary Notes ... **3**

Supplementary Note 1. Transformation of Scoring Matrix Using Match Bonus to be Comparable with SigAlign ... 3

Supplementary Note 2. Definition of SigAlign Alignment Results ... 3

Supplementary Note 3. Reproducing the SigAlign Algorithm via Dynamic Programming Method ... 4

Supplementary Note 4. Software Enhancements and Functionalities of SigAlign ... 5

Supplementary Note 5. Relationship of Magnitude Between $\boldsymbol{d2m}$ *and* $\boldsymbol{d2m-1}$ ... 7

Supplementary Note 6. K-mer Size Calculation Algorithm ... 8

Supplementary Note 7. Anchor Processing Order ... 9

Supplementary Note 8. Methodology for Profiling Memory Usage ... 9

Supplementary Note 9. Compilation and Usage of Demonstration Binary of SigAlign ... 10

Supplementary Note 10. Selection of Real Sequencing Datasets ... 11

Supplementary Note 11. Genome IDs in UHGG Database for Reference and Query ... 12

Supplementary Note 12. Methodology for Simulating Query ... 13

Supplementary Note 13. Method to Determine Correct Alignments ... 15

Supplementary Note 14. Calculations for Relative Abundance ... 16

Supplementary Note 15. Missed Alignments by BLASTn in Database Search ... 17

Supplementary Tables ... **18**

Supplementary Table 1. Parameters Impacting the Result in BWA-MEM and BLASTn ... 18

Supplementary Table 2. Versions and Commands for Compared Aligners ... 19

Supplementary Table 3. Commands Used in Demonstration Binaries of SigAlign ... 22

Supplementary Table 4. Gap-affine Penalties of Heuristic Aligners ... 23

Supplementary Table 5. Summary for Query Sequence Used in Tests ... 24

Supplementary Table 6. Information on Reference Sequences Including Building Time ... 25

Supplementary Table 7. Consistent Sensitivity Across Simulated Genomes ... 27

Supplementary Table 8. Detailed Values of RMSE and MAE in Metagenome Abundance Profiling ... 28

Supplementary Table 9. Detailed Values of False Negative Rate in Database Search Test ... 29

Supplementary Table 10. Reasons Behind the Reduction in Mean Memory Usage by Additional Threads ... 31

Supplementary Table 11. Performance of RazerS3 for Read Mapping Tasks ... 32

Supplementary Table 12. Precision in Simulated Short Reads Using Primary Alignment ... 34

Supplementary Figures ... **36**

Supplementary Figure 1. Iteration of Minimum Penalty Across Consecutive K-mers ... 36

Supplementary Figure 2. Relationship between $\boldsymbol{ln}$ *and* $\boldsymbol{pn}$ ... 37

Supplementary Figure 3. Metagenome Profiling Results for the Extended Database ... 39

Supplementary Figure 4. Database Search Results for Read-Mappers ... 40

Supplementary Figure 5. Time Series Memory Usage for All Tools ... 42

Supplementary Figure 6. Average Memory Usage Across Various Query Lengths ... 43

References ... **45**

Supplementary Notes

Supplementary Note 1. Transformation of Scoring Matrix Using Match Bonus to be Comparable with SigAlign

SigAlign employs a scoring function that utilizes three penalties: 1) mismatch penalty, (2) gap-open penalty, and (3) gap-extend penalty. An alternative form of a gap-affine penalty-based scoring function also includes a match bonus for paired bases. SigAlign can adapt to this scoring function by translating the value and interpretation.

Initially, the match bonus is integrated with penalties extending one base pair: 1) mismatch penalty and (2) gap-extend penalty. Subsequently, the maximum penalty per length (MaxP) is converted into the minimum score per length (MinS) using the following equation:

$$MinS = match\ bonus - \ MaxP$$

Given that the score utilizing the match bonus can be derived from the aligned length and penalties:

$$score = length * match\ bouns - penalty$$

For instance, when 1, 4, 6, and 1 are respectively applied as a match score, mismatch penalty, gap-open penalty, and gap-extension penalty, the transformed penalties become 5, 6, and 2 for mismatch, gap-open, and gap-extension penalties, respectively. Within this same scoring scheme, if 0.1 is applied as MaxP, then 0.9 (=1-0.1) corresponds to the minimum score per length.

Supplementary Note 2. Definition of SigAlign Alignment Results

SigAlign operates in two distinct modes: 1) Semi-global and 2) Local. The alignment results of these modes are referred to as "semi-global alignment" and "local alignment," respectively.

The semi-global alignment ensures that the starting point of the alignment aligns with the start of either the query or the target. Similarly, the end point of the alignment aligns with the end of either the query or the target. In contrast, local alignment does not impose conditions on the alignment location and may only cover a portion of both the query and target.

The semi-global alignment in SigAlign includes:

1. All semi-global alignments.
2. Selection of non-overlapping alignments in ascending penalty order.
3. Exclusion of alignments that do not meet the similarity cutoffs (MinL and MaxP).

The local mode alignment equals the semi-global alignment for every substring of the query. Therefore, the local mode alignment results always include the semi-global mode alignment results.

SigAlign outputs all alignments with non-overlapping positions. In SigAlign, "overlapping positions" are defined as the set of position pairs of connected (match or substitution) base pairs of the query and target. For example, in alignment result A, if the i-th base pair of the query and the j-th base pair of the target are connected, another alignment B sharing the same connected position pair (i, j) is considered overlapping with A. This aligns with the criteria used in alignment determination through the Dynamic Programming method, which evaluates whether to pass the match cell of the matrix.

The definition of overlapping positions lacks consensus across alignment algorithms. Therefore, we propose our own rationale. From an information content (entropy) perspective, our definition of overlapping positions is justified. Although the output of all possible alignment results satisfying the similarity cutoff may seem more intuitive from a data flow perspective, it leads to informational redundancy. For example, in a global alignment scenario involving 100 adenines (A) and 99 A, where one gap can appear in 100 potential positions along the entire sequence, all alignment results, barring specific operations, share the same alignment position, penalty, and length. Consequently, the joint entropy of alignment results A and B, H(A,B), is roughly equal to the individual entropy of either A or B (H(A) or H(B)). Hence, generating all 100 alignment results not only slows down the algorithm but also diminishes the informational value of each alignment.

SigAlign's alignment results "include" deduplicated results and become "identical" to such results following an additional deduplication process. The rationale for this operation lies in the fact that the impact of always using an additional deduplication process is negligible. Although it slightly reduces the algorithm's speed, cases requiring deduplication are infrequent. This disparity arises from the fact that deduplication based on the anchor (alignment starting point) does not always align with deduplication based on matched position pairs of the query and target. Nevertheless, the two deduplication methods usually yield the same results. Therefore, we have provided the option of obtaining perfectly deduplicated results through an additional process, while designing the default operation to always "include" deduplicated results.

Supplementary Note 3. Reproducing the SigAlign Algorithm via Dynamic Programming Method

SigAlign operates in two modes: semi-global and local. The protocol for achieving the semi-global mode alignment results through the Dynamic Programming (DP) method (1–3) is as follows:

1. Initiate the DP matrix, setting all margin (clipping) penalties to zero.
2. Populate the DP matrix.

3. Perform a backtrace, extracting the alignment result from each edge cell (i.e., cells situated in the final column or row of a matrix).
4. Organize the alignment results in optimal order (ascending penalty order).
5. In ascending order of optimism, output the results that meet the cutoff, excluding those overlapping with more optimal results (as detailed in the previous section).

While implementing the DP method, certain operational details are considered, such as the priority order of operations. Regardless of these particulars, the DP algorithm above produces operations nearly identical to SigAlign (the sequence of Match, Substitution, Insertion, and Deletion). To output the exact SigAlign results at both matrix and operation levels, the following rules are strictly adhered to:

- During DP matrix population, operations are prioritized in this order if the penalties are equal: 1) Match, 2) Deletion, 3) Insertion, 4) Substitution.
- At the backtrace stage, if the penalties are equal, Deletion and Insertion are prioritized over Substitution.
- When sorting alignment results, if penalties are equal, the query index of the edge cell is subsequently utilized in descending fsforder.

The procedure for achieving SigAlign's local mode results through the DP method is as follows:

1. Perform semi-global alignment for all substrings in the query.
2. Output the alignment for the larger length substring, ensuring no positional overlap.

In Local mode, if substring lengths are equal, the start index in the query from which the substring originates is prioritized.

There is an edge case where raw results from SigAlign and the DP method do not match perfectly. When there are numerous sub-optimal alignments—for instance, if the target possesses many repeat regions—SigAlign yields more alignment results than the DP method. SigAlign offers an additional deduplication function for alignment results; when SigAlign results undergo further deduplication using this function, they match the DP method outcomes. This function is not a default behavior because its thoroughness does not offset performance degradation resulting from additional function utilization. Even without this function, SigAlign ensures: if no alignment satisfies the cutoff, both SigAlign and the DP method yield no results. Importantly, for the most optimal alignment, SigAlign and DP method results are exactly equivalent. It's worth noting that SigAlign outputs results for all unique alignments, which must include DP method results.

Supplementary Note 4. Software Enhancements and Functionalities of SigAlign

This note delves deeper into the software enhancements and additional functionalities of SigAlign that were not extensively covered in the "Core Technical Advancements" of the main paper, aiming to maintain conciseness.

Initially, SigAlign leverages the FM-index to pinpoint patterns within multiple targets, as described in the "Determining Anchors (Step 1)" of "Detailed Algorithm Workflows" section. Broadly, sequence indices in biological alignment algorithms can be grouped into two main categories: 1) FM-index and 2) hashing (4). The FM-index is a sophisticated data structure, melding the Burrows-Wheeler transform (5) with an occurrence matrix, enabling efficient substring searches. In contrast, hashing converts varying-sized data into fixed-size values, generally facilitating faster data access through hash tables. Among the tools we assessed, BWA-MEM, HISAT2, and bowtie2 utilize FM-indices, whereas minimap2, BLASTn, and MMseqs2 adopt hash-based indexing. Although hashing generally outpaces the FM-index in terms of index generation and pattern localization (see reference building times in Supplementary Table 7), hash collisions can result in overlooked perfect match positions. To ensure strict adherence to similarity thresholds, SigAlign opts for the FM-index.

SigAlign utilizes a library known as "LT-FM-index", one of several types of FM-indices. The LT-FM-index employs a technique that uses a lookup table (LT) for the first k-mer of the occurrence matrix, allowing it to skip operations for the initial k-mers. Since the size of the LT increases exponentially with the length of the indexed k-mer, infrequent use of LT can lead to performance degradation due to speed reduction from data structure loading that surpasses the speed gain from using LT. However, SigAlign is particularly well-suited to using the LT-FM-index because 1) SigAlign always uses fixed-size k-mers, allowing it to consistently benefit from the first k-mer skip, and 2) SigAlign requires a relatively large number of pattern searches compared to other algorithms to produce non-heuristic results.

The concept of LT was inspired by the AvxWindowFmIndex library and its corresponding paper (6). Although AvxWindowFmIndex provides excellent performance, its focus is on speed improvements using AVX instructions. However, AVX is not essential for SigAlign, so it doesn't require AVX instructions. Additionally, SigAlign needed specific extra functionalities, such as the ability to handle non-nucleotide and non-amino acid sequences, integrate a serialization process directly into the reference object to streamline, and support various BWT sizes to adjust compression rates. Hence, we developed our own library, the LT-FM-index, for SigAlign's use. While its primary purpose is for integration with SigAlign, the LT-FM-index can also operate as a standalone library. It is an open-source library, crafted with the Rust programming language and under an MIT license. Its source code is accessible at the repository (https://github.com/baku4/lt-fm-index), and API documentation can be found in the library registry (https://docs.rs/lt-fm-index/).

SigAlign also introduces a specialized wavefront algorithm (WFA) (7) for anchor extension, as detailed in the "Extending Anchors (Step 2)" of "Detailed Algorithm Workflows" section. WFA employs a Breadth-First Search strategy for extensions with a gap-affine penalty, consuming less memory and achieving faster results than dynamic matrix computations. While a well-established WFA algorithm library exists (recently updated to WFA2), we re-engineered the WFA, incorporating features specifically tailored for SigAlign. One notable change involves incorporating data on deletion counts in each WFA component's structure padding space. For

effective anchor backtrace in SigAlign, it's essential to 1) select the correct backtrace starting point and 2) track all anchors traversed during backtrace. Consequently, determining the alignment length before initiating the backtrace from the endpoint, which is achievable by pre-identifying deletion or insertion counts, becomes imperative. A function to estimate the number of WFA components SigAlign might employ was also introduced.

The memory allocation approach in SigAlign is innovative. Not limited to WFA, memory space reuse extends to commonly used structures, like SigAlign symbols and alignment results. Memory allocation in SigAlign is distinct for the reference structure and the alignment workspace, as highlighted in the "Memory Footprint" (of "Results"). This separation facilitates efficient buffer management for frequently accessed structures. Consequently, SigAlign only allocates extra memory when managing larger structures.

Further, SigAlign incorporates numerous functionalities optimized for contemporary computing systems. For instance, SigAlign can specify a search range for pre-constructed reference sequences, eliminating the need to rebuild references for subsets. Another standout feature of SigAlign is its flexibility regarding sequence storage and pattern location indexing. While this study utilized in-memory storage, SigAlign's sequence repository can be a file or an external server. SigAlign is also versatile in build targets, supporting devices without SIMD instructions and can be constructed as WASM for web applications. A thorough exploration of SigAlign's array of features is available in the library's official documentation (https://docs.rs/sigalign/).

Supplementary Note 5. Relationship of Magnitude Between $\hat{d}_{2m}$ and $\hat{d}_{2m-1}$

Rather than verifying whether all values of $\hat{d}_n$ (penalty per length for n k-mers) are greater than $\bar{d}$ (cutoff: maximum penalty per length) in the main text, we have checked whether this is true for certain values of n. This was done by examining the relationship between $\hat{d}_{2m}$ and $\hat{d}_{2m-1}$, where there are no discontinuities in the formula.

The following are the derivations and simplifications of two cases where the relationship is clear:

- $\hat{d}_{2m+1} - \hat{d}_{2m} = \frac{m(k+1)(p_1-p_2)+p_1\left(2k+\frac{p_1}{p_e}-2\right)}{\left((2m+3)(k+1)+\frac{p_1}{p_e}-4\right)\left((2m+2)(k+1)+\frac{p_1}{p_e}-4\right)} > 0$
- $\hat{d}_{2m+2} - \hat{d}_{2m} = \frac{(p_1+p_2)\left(2k+\frac{p_1}{p_e}-2\right)}{\left((2m+4)(k+1)+\frac{p_1}{p_e}-4\right)\left((2m+2)(k+1)+\frac{p_1}{p_e}-4\right)} > 0$

Since $p_1 \geq p_2$, $k \geq 1$, and $m \geq 1$, both expressions are always greater than zero. Therefore, $\hat{d}_{2m+1}$ and $\hat{d}_{2m+2}$ are always greater than $\hat{d}_{2m}$. However, our derivations did not reveal whether $\hat{d}_{2m}$ is always greater than $\hat{d}_{2m-1}$. The method of simplifying this verification is further detailed in the main text.

Supplementary Note 6. K-mer Size Calculation Algorithm

ALGORITHM. CALCULATION OF PATTERN SIZE

Input: *$\bar{l}$ minimum length, $\bar{d}$ maximum penalty per length, p_e gap-extend penalty, p_1, p_2 penalties by pattern order*

Output: *k pattern size*

Function get_pattern_size($\bar{l}$, $\bar{d}$, p_e, p_1, p_2) **begin**
    $n \leftarrow 0$
    $k \leftarrow 0$
    $l \leftarrow \lceil (p_e(\bar{l} + 4) - p_1)/2p_e \rceil - 1$
    while ($k < l$) **do**
        $n \leftarrow n + 1$
        $u \leftarrow l - 1$ // Upper bound
        $l \leftarrow \lceil (p_e(\bar{l} + 4) - p_1)/2p_e \rceil - 1$ // Lower bound
        if ($n \; mod \; 2 = 1$) **then** // If *n* is odd
            $m \leftarrow (n + 1)/2$
            $k_1 \leftarrow \lceil (p_e(mp_1 + mp_2 - p_2) + \bar{d}(4p_e - p_1))/\bar{d}p_e(2m + 1) \rceil - 2$
            $k_2 \leftarrow \lceil (mp_e(p_1 + p_2) + \bar{d}(4p_e - p_1))/\bar{d}p_e(2m + 2) \rceil - 2$
            $k \leftarrow min(u, k_1, k_2)$
        else // If *n* is even
            $k \leftarrow min(u, k_2)$
        end if
    end while
return k;

Supplementary Note 7. Anchor Processing Order

For efficient processing, the extension and evaluation of anchors adhere to specific rules. The anchors are sorted in ascending order based on the position of 1) the query and 2) the target. Then, from left (front of the list) to right, proceed sequentially for each unskipped anchor (seed anchor):

1. If the seed anchor has not been extended, extend it.
2. Among the right traversed anchors of the seed anchor, perform the following steps in order from the rightmost traversed anchor to the leftmost traversed anchor:

    1) If the traversed anchor has not been extended, extend it.
    2) Determine whether the extension of left-side traversed anchors of the traversed anchor can be skipped.
    3) If the symbol of the traversed anchor includes a seed anchor, skip the traversed anchor as well.

3. If the alignment of the seed anchor satisfies the similarity cutoffs, print it out as a result.

The evaluation is conducted sequentially in an ordered list, and the extension may not be sequential depending on whether an anchor has been traversed by another anchor.

Supplementary Note 8. Methodology for Profiling Memory Usage

Memory consumption over time was monitored using a memory profiler for each tool. The initial step was to establish a baseline by determining each command's execution time without memory profiling. This was necessary, as memory profiling can lengthen execution times due to the constant monitoring of memory allocations and deallocations.

For this purpose, we employed mprof, the executable binary for the "memory_profiler" package, available at "https://github.com/pythonprofilers/memory_profiler". We used version 0.61.0 of the "memory_profiler" package. The mprof command was executed as follows:

```
mprof run --include-children \
          --interval 0.001 \
          "${alignment_command_of_each_tool}"
```

Here, "alignment_command_of_each_tool" pertains to the specific alignment command associated with each tool.

Supplementary Note 9. Compilation and Usage of Demonstration Binary of SigAlign

For convenient testing, we developed a command-line demonstrative binary that utilizes SigAlign for alignment tasks. The binary we used in our tests was developed using version 0.3.2 of SigAlign. This binary can be built from the source code available at SigAlign's Zenodo (https://doi.org/10.5281/zenodo.10253841) using the following commands:

```
cargo build --release -p sigalign-demo-aligner
```

The built 'sigalign-demo-aligner' binary has two subcommands: 'reference' and 'alignment'. 'reference' takes one FASTA file as input to build a reference and outputs it. 'alignment' performs local alignment using one FASTA file as a query and a reference file built by 'reference' as the target. Detailed usage can be checked by running 'sigalign-demo-aligner' (without parameters or with the --help parameter).

The output of the tool is in TSV format (with TAB as the separator) and contains the following 11 columns: query_label, is_forward, reference_index, target_index, penalty, length, start_index_of_query, end_index_of_query, start_index_of_target, end_index_of_target, operations. To elaborate on a few columns, the 'is_forward' value is 1 for forward and 0 for reverse complementary. The indexes are all zero-based. The 'reference_index' is for when the reference is divided into multiple chunks. 'target_index' is the index of the target within a reference chunk. 'operations' is a concatenated string that describes the sequence of alignment operations. Each segment consists of an operation type (Match, Substitution, Insertion, Deletion) followed by the number of times that operation occurs consecutively. For example, 'M12S1D3' signifies 12 matches, followed by 1 substitution, and then 3 deletions. Note that this differs from the SAM format's CIGAR string.

Please note that SigAlign is primarily intended to be used as a library, and this demonstration binary is written for testing purposes, so it lacks several features.

The multi-threaded version used in our memory profiling can be built by adding the 'thread' feature to the build command:

```
cargo build --release --features thread \
-p sigalign-demo-aligner
```

The multi-threaded version accepts multiple FASTA files as input in the 'alignment' subcommand and processes them in separate threads.

Note that the following describes the 0.3.1 version (https://doi.org/10.5281/zenodo.10049389), which is no longer in use due to compiler issues. Though we have transitioned to using version 0.3.2 throughout the revision process, we provide a memo on issues and solutions for the 0.3.1

version, which remains accessible on Zenodo for those who might inadvertently or intentionally utilize it. The build command for the 0.3.1 version is as follows:

```
# Defining the specific version of Rust compiler
rustup override set 1.69.0

# 'clap' crate is needed to be defined with precise version
cargo update -p clap@4.4.11 --precise 4.0.26

# Build sigalign-demo-aligner
cargo build --release -p sigalign-demo-aligner
```

These build commands include specifying the compiler version to resolve the issue of SigAlign version 0.3.1 not being compatible with the latest Rust compiler. The behavior of SigAlign version 0.3.1 depends on the 'rustc' version as follows:

- From version 1.57.0 up to 1.69.0 (as of 2023-04-16): Works correctly.
- From version 1.70.0 up to 1.73.0: Compiles but SigAlign may produce unexpected results.
- From version 1.74.0: Compilation errors occur.

When initially tested for the manuscript, the the version of rustc was below 1.69.0, so our code worked without issues. However, as the compiler version increased, some of the code we used conflicted with the latest compiler. We have completely resolved this issue in SigAlign version 0.3.2. SigAlign 0.3.2 works correctly with all the latest compilers.

We strongly recommend using version 0.3.2 instead of 0.3.1. The fact that SigAlign version 0.3.1 builds incompletely without any error messages from rustc version 1.70.0 to 1.73.0 is a significant concern. Furthermore, we cannot predict how the external libraries we used will be updated in the future. As of the current writing date (December 12, 2023), building version 0.3.1 with the above commands is possible, but precise version specification for other external libraries might be required later. Therefore, version 0.3.2 is more stable for building and operation.

Supplementary Note 10. Selection of Real Sequencing Datasets

In selecting sequencing datasets, our aim was to capture the varied nature of read data, hence we sourced data from multiple sequencing platforms. We structured our tests into two primary categories to manage the complexity: 1) alignment of short-reads to a bacterial genome, and 2) alignment of long-reads to a human genome. *Mycobacterium tuberculosis* was chosen as the bacterial genome because of its widespread use in Whole Genome Sequencing (WGS) for diagnosis and public health monitoring (8).

For short-read data, we selected the NovaSeq and MiSeq platforms from Illumina. For long reads, we chose the MinION from Oxford Nanopore Technologies (ONT) and the Sequel II from Pacific Biosciences (PacBio). To capture the advancements in sequencing technology, we

specifically targeted datasets generated by relatively new techniques, such as preferring NovaSeq over HiSeq from Illumina. The decision to use the R 10.4 flowcell for ONT's MinION was influenced by its higher accuracy (9). We also included HiFi reads from PacBio.

Each dataset was carefully curated to ensure consistency. Initially, data for all platforms were sourced from individual BioProjects, identified by their respective NCBI/EBI accession numbers. Specifically, the accession numbers for NovaSeq, MiSeq, MinION, and Sequel II are PRJEB49562, PRJEB35201, PRJNA875576, and PRJNA529679. While short-reads underwent basic quality control and adapter trimming via “fastp” tool (10), the long-read datasets remained unprocessed.

The sequence reads from each project were pooled together and then filtered to ensure the read lengths fell within a specific range. This pre-filtering is necessary as outliers can significantly impact the overall processing speed, and such effects would go undetected in our tests. Consider a sample with most reads at 1000bp, interspersed with a few at 100,000bp. Tools like SigAlign are sensitive to read length when it comes to throughput. Consequently, processing bottlenecks for 100,000bp reads would disproportionately affect the total speed, regardless of the throughput for 1000bp reads. Ideally, we would calculate per-read speed, allowing for a median-like measure less sensitive to outliers. However, as most tools process reads in bulk (FASTA format in our tests), we were limited to calculating average processing times. Limiting the read length range, therefore, provides a less biased speed measurement.

To include the majority of read lengths, we determined ranges that encompass the average read lengths for each sequencing pool. The recorded average read lengths for NovaSeq, MiSeq, MinION, and Sequel II were 97.62, 283.63, 3,767.05, and 13,478.34 bp, respectively. The set read length ranges for these platforms were 90~101, 250~301, 3,000~4,000, and 12,000~14,000 bp.

These ranges include the median values (101, 300, 13,382 bp) for NovaSeq, MiSeq, and Sequel II, respectively. For MinION, we determined that the distribution of read lengths does not follow a normal distribution and is skewed to the left (with the mode in the 700~900 bp range), thus the median is less informative. Consequently, whether the range includes the median (1,879 bp) or the third quartile (3,809 bp), the effect is minimal. We therefore chose to use the average length to assess performance at higher lengths.

From the range-restricted sequence pool of each platform, reads were then extracted at regular intervals, targeting a specific number of reads: 10 million for NovaSeq and MiSeq, 200,000 for MinION, and 100,000 for Sequel II. These selected reads were combined into a single FASTA file, which was then employed as the test query sequence.

Supplementary Note 11. Genome IDs in UHGG Database for Reference and Query

For the "Human Gut Metagenome Abundance Profiling" and "Recall of Alignment Results in Large Database" tests in the "Application in Database Search" section, as well as the "Memory Footprint" section, reference sequences were sourced from the UHGG (11) database. To enable precise reproduction of these sequences, we have provided the corresponding genome IDs from

the UHGG database. These IDs can be accessed from the following GitHub Gist: https://gist.github.com/baku4/a48c86b6033216a2a446bbff75babc69.

The provided Gist contains:

- For the "Human Gut Metagenome Abundance Profiling" test:
  - "GenomeID_Info_AbundanceProfiling.csv" – This file provides the IDs of genomes used in the "Human Gut Metagenome Abundance Profiling" test. It includes details on the Genome ID, PrevalenceScore, taxonomical classification (Phylum, Class, Order, Family, Genus, Species), and genomic attributes (Length, N_contigs, N50, GC_content) for each genome. The PrevalenceScore values are extracted from the HumGut (12) database (“prevalence_score” column in HumGut). The table is sorted in descending order of PrevalenceScore, with indices up to 100 used in the main paper's "restricted database," up to 200 for the "full database," and up to 400 (the entire table) for the "extended database (Supplementary Figure 3).”
- For the "Recall of Alignment Results in Large Database" test:
  - "GenomeID_Index_DatabaseSearch.csv" - This file enumerates indices and the corresponding Genome IDs that are used in the "Recall of Alignment Results in Large Database" test. The primary purpose of this file is to provide a reference index that is utilized in the following "IndexList_Ref_DatabaseSearch.csv" file.
  - - "IndexList_Ref_DatabaseSearch.csv" - This file lists an index for each reference used in the "Recall of Alignment Results in Large Database" test. Each line of the file represents a unique quantity of genomes and a pipe-separated ('|') index list that refers back to the indices provided in the "GenomeID_Index_DatabaseSearch.csv" file.
- For "Memory Footprint" section
  - "GenomeID_Info_MemoryFootprint.csv" - This file contains detailed information on all genomes merged into a single reference for the "Memory Footprint" test. It provides data on the Genome ID, taxonomical classification (Phylum, Class, Order, Family, Genus, Species), and genomic attributes (Length, N_contigs, N50, GC_content) of each genome.

These files collectively contain all necessary information to reproduce the results obtained in the paper using the same references.

Supplementary Note 12. Methodology for Simulating Query

In our tests, all tests except for the "Performance for Diverse Sequencing Platforms" part in the "Read Mapping Across Various Sequencing Data" section use simulated queries. The sections or subsections of the Results using simulated queries can be broadly categorized into four types:

1) "Accuracy in Simulated Short Reads Across Organisms" subsection (within the "Read Mapping Across Various Sequencing Data" section)
2) "Human Gut Metagenome Abundance Profiling" subsection (within the "Application in Database Search" section)

3) "Recall of Alignment Results in Large Database" subsection (within the "Application in Database Search" section)
4) "Memory Footprint" section

Firstly, for the "Accuracy in Simulated Short Reads Across Organisms" test, we utilized Mason (13) (version 2.0.9) known for most accurately replicating Illumina platforms' data (14).

Secondly, in the "Human Gut Metagenome Abundance Profiling" test, InSilicoSeq (15) (version 1.6.0) was used, which is specialized in simulating metagenomic sequences.

Thirdly, for the "Recall of Alignment Results in Large Database" test, sequences were manually extracted without using a tool. This approach was taken to better reflect the search for sequences with the same level of similarity (same at the species level but different at the strain level) in a large database. We used a sliding window method to manually extract queries. Within the UHGG database, a 300bp sequence from the "MGYG000002506" genome (not included in any reference), representative of an E. coli genome, was extracted, with its position shifted by 11bp.

Fourthly, for the "Memory Footprint" section, dwgsim (available at https://github.com/nh13/DWGSIM, version 0.1.13), which allows for flexible setting of target sequence lengths, was employed to extract sequences of various lengths.

Commands for the Mason, InSilicoSeq, and dwgsim tools are as follows:

Mason:

```
mason_simulator --seed 0 -n "${query_count*2}" \
                --num-threads 8 \
                --illumina-read-length "${query_length}" \
                --fragment-mean-size "${query_length*2}"\
                -ir "${fasta_of_target}" \
                -o "${output_path(.fastq)}" \
                -oa "${output_path(.sam)}"
```

InSilicoSeq:

```
iss generate --seed 0 -n "${query_count*2}" \
                --model MiSeq \
                --cpus 6 \
                --draft "${fasta_of_target}" \
                --abundance_file "${abundance_file}" \
                --output "${output_path}"
```

dwgsim:

```
dwgsim -N "${query_count*2}" -z 0 -y 0.01 -H \
          -1 "${query_length}" -2 0 \
          "${fasta_of_target}" "${output_path}"
```

In these commands, "fasta_of_target" is the path to the reference FASTA file, and "output_path" is the output destination. "query_length" and "query_count" indicates the desired query length and count, respectively. During simulation, it was noted that the actual output sometimes differed from the input parameters. To address this, a pool containing double the intended query count was created. In the "abundance_file" used by InSilicoSeq, each genome in the "fasta_of_reference" was allocated an equal proportion (0.005=1/200). If multiple contigs of different lengths were present for each genome, the total proportion was divided according to the length of the contigs (for example, if there are 4Mbp and 1Mbp contigs, the 4Mbp receives 0.004 and the 1Mbp receives 0.001). The simulated reads were randomly extracted and combined into one FASTA file, which was then used as the query.

Supplementary Note 13. Method to Determine Correct Alignments

We considered an alignment to be "correct" if it covered the simulated position on the same genome and strand direction. Specifically, if the alignment covered indices a_1 to a_2 on the reference and the simulated position was from s_1 to s_2, the correctness was defined as:

$$(a_1 \leq s_2)\ and\ (a_2 \geq s_1)$$

For example, if a query originated from 100-400 bp on the reference, alignments mapped to 80-380 bp or 120-420 bp would be considered correct.

This criterion was used to allow slight differences due to minor algorithmic decisions, ensuring a fairer evaluation. For instance, consider the following homopolymer example (query: 5 "A"s, reference: 6 "A"s, "-" is gap):

- Ground truth from simulator (mapped to 1-5 bp):
    - AAAAAA (reference)
    - AAAAA- (query)
- Alignment result (mapped to 2-6 bp):
    - AAAAAA (reference)
    - -AAAAA (query)

In this case, the alignment result would differ from the ground truth for all matched base pairs due to a single offset. Determining correctness only when all positions in the alignment exactly matched would introduce a bias, making alignments in the above case appear more similar to incorrect ones rather than correct ones. Therefore, we allowed differences resulting from such minor algorithmic decisions.

Supplementary Note 14. Calculations for Relative Abundance

This note describes the methodology used to calculate relative abundance in the tests performed in the "Human Gut Metagenome Abundance Profiling" subsection of the "Application in Database Search" section in the main paper.

Relative abundance was calculated using the results from mapping a query, consisting of 50 million reads, to a database, that is, the reference. Since each read could be aligned to multiple results, we selected the single best-matching genome for each read and then calculated the relative abundance for each genome by dividing the number of reads mapped to that genome by the total number of aligned reads.

Let:

- A_X = The number of reads whose best mapping is to genome X
- A_T = The total number of reads that have alignments in the dataset

Then, the relative abundance (RA) of genome X can be calculated using the formula:

- $RA_X = \frac{A_X}{A_T}$

Note that there can be reads without any alignment results, hence the A_T may differ from the total number of reads (50 million): $A_T = \sum A_X \leq 50M$. The sum of the relative abundances always equals to 1.

Next, we describe the method used to select the "best-matching" genome. The tools we employed include SigAlign, two database search tools (BLASTn, MMseqs2), and four read mappers (bowtie2, BWA-MEM, HISAT2, minimap2):

- SigAlign: The alignment with the longest query length is considered optimal. If alignments have equal lengths, the one with the smaller penalty is considered optimal.
- Database search tools: A smaller E-value indicates a more optimal result.
- Read mappers: Only primary alignments are used. Each read has, at most, one primary alignment if any alignment is present.

E-value and primary alignments are the most commonly used indicators of alignment significance in their respective domains.

For SigAlign, BLASTn, and MMseqs2, if there are ties (i.e., alignments with equal query length and penalty for SigAlign, or equal E-values for database search tools), a random selection is made. The method for random selection is as follows:

- Using Python's "numpy" library, the random seed is initialized with the `seed` method, and pseudo-random numbers are assigned to each record in sequence using the `rand`

method:

```
np.random.seed(seed)
np.random.rand(number_of_lines_in_tsv)
```

- Among the tied alignments, the one with the highest assigned pseudo-random number is selected.
- For SigAlign, BLASTn, and MMseqs2, the RMSE and MAE were measured 50 times with different seeds (seed values from 0 to 49), and the average values are reported (detailed values are in Supplementary Table 8).

Specifically, in the main text, panel (a) of Figure 3 (relative abundance) uses results with seed 0, while panel (b) (RMSE) shows the average RMSE values calculated from seeds 0 to 49.

Supplementary Note 15. Missed Alignments by BLASTn in Database Search

In database searches involving larger references ("Recall of Alignment Results in Large Database" in main paper), BLASTn occasionally fails to detect alignments present in smaller references. Specifically, when aligning to references comprised of 16, 256, 1024, and 4096 strains, BLASTn overlooked 1, 4, 2, and 113 alignments respectively when compared to their half-sized references. Comprehensive data pertaining to these omitted alignments is provided. However, due to the extensive nature of the information, it is not feasible to incorporate directly within the main text. Instead, the table elucidating the missed alignments is available at the following GitHub Gist under the file "MissedAlignments_BLASTn_DatabaseSearch.csv": https://gist.github.com/baku4/a48c86b6033216a2a446bbff75babc69. This table delineates the locations of missed alignments in both the larger reference and its half-sized reference, the strain count, the specific contig and position from which the query originated, and the alignment's positions in both the query and target. Positions are denoted using a 1-based system, mirroring the convention used in BLASTn's outputs.

Supplementary Tables

Supplementary Table 1. Parameters Impacting the Result in BWA-MEM and BLASTn

	BWA-MEM		BLASTn	
	**Count**	**Parameters**	**Count**	**Parameters**
**Influential Parameters**	15	-k | -w | -d | -r | -y | -c | -D | -W | -A | -B | -O | -E | -L | -T | -a	22	-evalue | -word_size | -gapopen | -gapextend | -penalty | -reward | -num_descriptions | -num_alignments | -dust | -soft_masking | -max_hsps | -culling_limit | -best_hit_overhang | -best_hit_score_edge | -subject_besthit | -max_target_seqs | -xdrop_ungap | -xdrop_gap | -xdrop_gap_final | -min_raw_gapped_score | -ungapped | -window_size
**Neutral Parameters**	21	-t | -m | -S | -P | -U | -x | -p | -R | -H | -o | -j | -5 | -q | -K | -v | -h | -C | -V | -Y | -M | -I	46	-query | -query_loc | -strand | -task | -db | -out | -use_index | -index_name | -subject | -subject_loc | -outfmt | -show_gis | -line_length | -html | -sorthits | -sorthsps | -filtering_db | -window_masker_taxid | -window_masker_db | -lcase_masking | -gilist | -seqidlist | -negative_gilist | -negative_seqidlist | -taxids | -negative_taxids | -taxidlist | -negative_taxidlist | -entrez_query | -db_soft_mask | -db_hard_mask | -perc_identity | -qcov_hsp_perc | -template_type | -template_length | -dbsize | -searchsp | -sum_stats | -import_search_strategy | -export_search_strategy | -no_greedy | -off_diagonal_range | -parse_deflines | -num_threads | -mt_mode | -remote

The table presents the effects of various parameters on alignment results for BWA-MEM and BLASTn. Results are categorized based on whether they produce the same alignment - defined as all queries aligning to the same location on an identical target. 'Influential Parameters' represent those that alter alignment outcomes. Conversely, 'Neutral Parameters' refer to those that do not modify the alignment outcomes. This category comprises parameters potentially affecting the alignment but unused in this analysis (e.g., parameters relevant to paired-end alignment), as well as parameters unrelated to the algorithm (e.g., parameters altering the format or adding a header line).

Supplementary Table 2. Versions and Commands for Compared Aligners

Tool	Version	Command to Build Reference	Settings	Command to Perform Alignment
BLASTn	2.12.0	makeblastdb \ -in "${fasta_of_target}" \ -dbtype nucl	Default	blastn -query "${fasta_of_query}" \ -db "${fasta_of_target}" \ -outfmt 6 \ > "${result_file}"
			Print All	blastn -query "${fasta_of_query}" \ -db "${fasta_of_target}" \ -max_target_seqs 10000000 \ -outfmt 6 \ > "${result_file}"
MMseqs2	13.45111	mmseqs createdb \ "${fasta_of_target}" \ "${target_db_file}" mmseqs createdb \ "${fasta_of_query}" \ "${query_db_file}"	Default	mmseqs search --threads 1 \ --search-type 3 \ --remove-tmp-files 1 \ "${query_db_file}" "${target_db_file}" \ "${result_db_file}" "${tmp_dir}" mmseqs convertalis "${query_db_file}" \ "${target_db_file}" \ "${result_db_file}" "${result_file}"
			Print All	mmseqs search --threads 1 \ --search-type 3 \ --remove-tmp-files 1 \ --max-seqs 10000000 \ "${query_db_file}" "${target_db_file}" \ "${result_db_file}" "${tmp_dir}" mmseqs convertalis "${query_db_file}" \ "${target_db_file}" \ "${result_db_file}" "${result_file}"
BWA-MEM	0.7.17-r1188	bwa index "${fasta_of_target}"	Default	bwa mem "${fasta_of_target}" \ "${fasta_of_query}" \ > "${result_file}"
			Print All	bwa mem -a "${fasta_of_target}" \ "${fasta_of_query}" \ > "${result_file}"
Bowtie2	2.4.5	bowtie2-build "${fasta_of_target}" \ "${fasta_of_target}"	Default	bowtie2 --local -f \ -x "${fasta_of_target}" \ -U "${fasta_of_query}" \ > "${result_file}"
			Print All	bowtie2 -a --local -f \ -x "${fasta_of_target}" \ -U "${fasta_of_query}" \ > "${result_file}"

HISAT2	2.2.1	`hisat2-build "${fasta_of_target}" \`<br>`    "${fasta_of_target}"`	Default	`hisat2 -f \`<br>`    -x "${fasta_of_target}" \`<br>`    -U "${fasta_of_query}" \`<br>`    > "${result_file}"`
			Print All	`hisat2 --all --secondary -f \`<br>`    -x "${fasta_of_target}"\`<br>`    -U "${fasta_of_query}" \`<br>`    > "${result_file}"`
Minimap2	2.24-r1122	`minimap2 -t 1 \`<br>`    -d "${reference_file}" \`<br>`    "${fasta_of_target}"`	Default	`minimap2 -t 1 \`<br>`    -a "${reference_file}" \`<br>`    "${fasta_of_query}" \`<br>`    > "${result_file}"`
			Print All	`minimap2 -t 1 \`<br>`    -N 2147483647 \`<br>`    -a "${reference_file}" \`<br>`    "${fasta_of_query}" \`<br>`    > "${result_file}"`

This table specifies the versions and command syntax for the alignment tools used in our evaluation. The first and second columns list the names of the tools and their version specifications, respectively. The third column shows the commands employed for pre-building the reference. The fourth column illustrates the operational settings, and the fifth column details the commands used for alignments for each setting. There are two operational settings: 'Default', which is the standard setting that does not modify parameters affecting the results, and 'Print All', a setting designed to provide all alignment positions.

More specifically, the 'Default' settings do not adjust the output of alignments, standardizing only the number of threads and output formats for a fair performance evaluation. 'Default' options limit the number of alignments for database search tools, whereas they output only primary alignments for read mappers. Consequently, we specified an additional 'Print All' option for scenarios where all alignment positions need to be reported. For database search tools under 'Print All', we simply set the output limit to a very high number, 10 million, and for read mappers, we utilized special options that enable the tool to output all secondary alignments as well.

Here is how 'Default' and 'Print All' are used in the paper:

- 'Default': In the main paper, 'Default' options were used for all tests except for the "Recall of Alignment Results in Large Database" test (in the "Application in Database Search" section).
- 'Print All': This setting was only used in the "Recall of Alignment Results in Large Database" test in the main paper. 'Print All' commands are provided even for tools not featured in the main paper because the results using 'Print All' for all tools are available in the supplementary materials (Supplementary Table 9, Supplementary Figure 4).

The utilized variables are defined as follows: “fasta_of_target” indicates the path to the FASTA file containing the reference target sequences; “reference_file” specifies the path to the reference file; “fasta_of_query” represents the paths to the FASTA files serving as queries; and “result_file” establishes the path for the output results file. For BLASTn, the output format is TSV instead of SAM because in the tested version of BLASTn, outputting many alignment results in SAM format can lead to a significant slowdown and sometimes freezing issues. TSV format provides a more precise indicator for E-value and is a common format that is also consistent with MMseqs2's output format.

Supplementary Table 3. Commands Used in Demonstration Binaries of SigAlign

Test Name	Commands	
	**Building Reference**	**Performing Alignment**
Read Mapping	`sigalign_demo_binary reference \`<br>`    --input "${fasta_of_target}" \`<br>`    --output "${reference_file}"`	`sigalign_demo_binary alignment \`<br>`    --input "${fasta_of_query}" \`<br>`    --reference "${reference_file}" \`<br>`    --penalties "${px}" "${po}" "${pe}" \`<br>`    --cutoffs "${min_len}" "${max_ppl}" \`<br>`    > "${result_file}"`
Database Search	`sigalign_demo_binary reference \`<br>`    --input "${fasta_of_target}" \`<br>`    --divide 3700000000 \`<br>`    --output "${reference_file}"`	
Memory Footprint	`sigalign_demo_binary reference \`<br>`    --input "${fasta_of_target}" \`<br>`    --divide "${chunk_size}" \`<br>`    --output "${reference_file}"`	
Memory Footprint<br>(with multiple threads)		`sigalign_demo_binary_thread alignment \`<br>`    --input "${fasta_of_queries[@]}" \`<br>`    --reference "${reference_file}" \`<br>`    --thread "${thread}" \`<br>`    --penalties "${px}" "${po}" "${pe}" \`<br>`    --cutoffs "${min_len}" "${max_ppl}" \`<br>`    > "${result_file}"`

This table enumerates all the commands used within the SigAlign demo binary for testing. The variables are defined as follows: "fasta_of_target "specifies the path to the FASTA file containing the target sequences used as references, "reference_file" denotes the path to the reference file, "chunk_size" represents the maximum size of a single chunk in base pairs (bp), "fasta_of_query" and "fasta_of_queries" correspond to the paths to the FASTA files used as queries for single and multiple files respectively, "px", "po", and "pe" are the penalties for mismatch, gap-opening, and gap-extension respectively, "min_len" and "max_ppl" define the similarity cutoffs including the minimum alignment length and the maximum penalty per alignment length respectively, "result_file" specifies the path to the file where the results will be stored, and "thread" denotes the number of threads to be used.

Supplementary Table 4. Gap-affine Penalties of Heuristic Aligners

Tools	Gap-Affine Penatlies with Match Bonus			
	Match Bonus	Mismatch Penalty	Gap-open Penalty	Gap-extend Penalty
SigAlign (*strictest*, *strict*, *lenient*)	0	4	6	2
BLASTn	1	3	5	2
MMseqs2	2	3	5	2
BWA-MEM	1	4	6	1
Bowtie2	2	6	5	3
Hisat2	0	6	5	3
Minimap2	2	4	4	2

All the tools tested in this study employ gap-affine penalties as their scoring matrix. This table details the scoring matrix values for each high-throughput aligner. It's worth noting that a scoring matrix which utilizes a match bonus can be integrated into SigAlign through a translation process (refer to Supplementary Note 1).

Supplementary Table 5. Summary for Query Sequence Used in Tests

Section	Subsection	Query Name	Number of Sequences	Min Length	Avg Length	Max Length
Read Mapping Across Various Sequencing Data	Performance for Diverse Sequencing Platforms	Illumina NovaSeq	10,000,000	90	100.9	101
		Illumina MiSeq	10,000,000	250	296.5	301
		ONT MinION	200,000	3,000	3,458.2	4,000
		PacBio Sequel II	100,000	12,000	13,060.5	14,000
	Accuracy in Simulated Short Reads Across Organisms	Simulated from *M. tuberculosis*	10,000,000	300	300.0	300
		Simulated from *E. coli*	10,000,000	300	300.0	300
		Simulated from *S. cerevisiae*	5,000,000	300	300.0	300
		Simulated from *A. thaliana*	5,000,000	300	300.0	300
		Simulated from *D. melanogaster*	5,000,000	300	300.0	300
		Simulated from *O. sativa*	5,000,000	300	300.0	300
		Simulated from *D. rerio*	1,000,000	300	300.0	300
		Simulated from *M. musculus*	1,000,000	300	300.0	300
		Simulated from *H. sapiens*	1,000,000	300	300.0	300
Application in Database Search	Human Gut Metagenome Abundance Profiling	Simulated from top 200 genera	50,000,000	301	301.0	301
	Recall of Alignment Results in Large Database	Extracted from MGYG000002506	485,353	300	300.0	300
Memory Footprint		Simulated 250 bp	1,000,000	250	250.0	250
		Simulated 500 bp	1,000,000	500	500.0	500
		Simulated 1000 bp	1,000,000	1,000	1,000.0	1,000
		Simulated 2000 bp	1,000,000	2,000	2,000.0	2,000
		Simulated 4000 bp	1,000,000	4,000	4,000.0	4,000

This table presents basic statistics for each FASTA file used as query sequences in testing. All queries are consolidated into a single FASTA file per test to minimize file loading overhead.

Supplementary Table 6. Information on Reference Sequences Including Building Time

Reference Sequences						Reference Generation Time (Hour:Minute:Second)						
						LF-mapping (FM-index)				Hashing (Hash table)		
**Section**	**Subsection**	**Reference name**	**Organism**	**Num. contigs**	**Reference size (bp)**	**SigAlign**	**Bowtie2**	**BWA-MEM**	**HISAT2**	**Minimap2**	**BLASTn**	**MMseqs2**
Read Mapping Across Various Sequencing Data	Performance for Diverse Sequencing Platforms	H37Rv	*Mycobacterium Tuberculosis*	1	4,411,532	0:00:00.6	0:00:04.6	0:00:02.4	0:00:03.1	0:00:00.4	0:00:00.2	0:00:00.1
		T2T-CHM13v2.0	*Homo sapiens*	25	3,117,292,070	0:15:47.4	2:21:28.8	1:02:39.0	1:25:19.3	0:02:51.4	0:00:25.5	0:00:26.9
	Accuracy in Simulated Short Reads Across Organisms	Mtb	*Mycobacterium tuberculosis*	1	4,411,532	0:00:00.6	0:00:04.7	0:00:02.5	0:00:03.2	0:00:00.4	0:00:00.2	0:00:00.1
		Ecoli	*Escherichia coli*	3	5,594,605	0:00:00.8	0:00:05.7	0:00:02.9	0:00:03.7	0:00:00.5	0:00:00.2	0:00:00.1
		Yeast	*Saccharomyces cerevisiae*	17	12,157,105	0:00:01.9	0:00:14.5	0:00:07.8	0:00:08.1	0:00:01.0	0:00:00.3	0:00:00.2
		ThaleCress	*Arabidopsis thaliana*	7	119,668,634	0:00:24.7	0:03:08.1	0:01:43.6	0:01:49.7	0:00:08.0	0:00:01.4	0:00:01.5
		FruitFly	*Drosophila melanogaster*	1,870	143,726,002	0:00:29.2	0:05:00.0	0:02:05.4	0:02:55.0	0:00:09.3	0:00:01.9	0:00:00.8
		Rice	*Oryza sativa*	58	374,422,835	0:01:21.1	0:12:39.2	0:06:10.5	0:07:10.7	0:00:23.0	0:00:04.0	0:00:03.9
		Zebrafish	*Danio rerio*	1,923	1,679,203,469	0:06:48.6	1:22:37.4	0:34:14.3	0:46:39.7	0:01:36.9	0:00:18.1	0:00:10.2
		Mouse	*Mus musculus*	61	2,728,222,451	0:12:35.0	2:18:13.3	0:59:15.4	1:13:53.0	0:02:30.8	0:00:33.5	0:00:13.9
		Human	Homo sapiens	24	3,117,275,501	0:15:47.4	2:25:56.4	1:11:03.0	1:33:48.4	0:02:49.7	0:00:24.5	0:00:24.4
Application in Database Search	Human Gut Metagenome	Restircted database	Metagenome	9,942	269,638,443	0:00:59.5	0:07:08.9	0:04:14.7	0:04:25.9	0:00:16.3	0:00:02.5	0:00:01.0
		Full database	Metagenome	19,515	521,974,539	0:02:11.0	0:15:56.5	0:08:58.2	0:08:46.1	0:00:31.6	0:00:04.7	0:00:01.9

	Abundance Profiling	Extended database	Metagenome	41,860	1,004,190,874	0:04:12.0	0:31:07.7	0:17:39.2	0:17:21.4	0:00:52.3	0:00:08.4	0:00:03.6
	Recall of Alignment Results in Large Database	1 strain	*Escherichia coli*	42	4,723,963	0:00:00.9	0:00:04.4	0:00:03.5	0:00:02.8	0:00:00.4	0:00:00.7	0:00:00.2
		2 strains		78	8,219,739	0:00:01.6	0:00:11.7	0:00:05.0	0:00:06.9	0:00:00.7	0:00:00.7	0:00:00.2
		4 strains		754	17,039,784	0:00:03.0	0:00:31.6	0:00:10.1	0:00:18.6	0:00:01.1	0:00:00.8	0:00:00.2
		8 strains		1,702	33,870,919	0:00:06.4	0:01:05.8	0:00:25.0	0:00:39.0	0:00:02.1	0:00:00.9	0:00:00.3
		16 strains		4,057	68,493,160	0:00:12.7	0:03:20.3	0:00:58.6	0:01:44.3	0:00:03.8	0:00:01.0	0:00:00.3
		32 strains		8,813	137,501,350	0:00:27.0	0:09:52.4	0:02:05.7	0:04:58.3	0:00:07.1	0:00:01.8	0:00:00.6
		64 strains		14,797	274,553,581	0:00:53.8	0:34:13.8	0:04:51.3	0:17:19.7	0:00:13.5	0:00:03.0	0:00:01.1
		128 strains		34,716	552,900,929	0:01:53.5	1:49:10.2	0:09:47.9	0:53:59.1	0:00:35.0	0:00:05.7	0:00:02.0
		256 strains		64,095	1,133,363,199	0:04:08.1	4:31:00.4	0:22:04.9	2:13:46.3	0:01:07.5	0:00:11.2	0:00:04.5
		512 strains		133,438	2,264,073,305	0:09:10.3	10:07:30.1	0:50:49.9	5:05:31.5	0:02:19.4	0:00:20.9	0:00:07.8
		1024 strains		269,366	4,516,629,383	0:19:31.3	23:06:47.5	1:39:15.3	11:38:31.2	0:04:26.1	0:00:41.6	0:00:13.9
		2048 strains		553,143	8,993,241,831	0:42:22.2	49:24:42.2	4:18:41.4	24:38:46.2	0:08:29.3	0:01:23.5	0:01:03.4
		4096 strains		1,083,955	18,011,785,342	1:20:25.0		10:30:02.4	66:42:12.9	0:17:28.6	0:02:42.2	
Memory Footprint		Metagenome	Metagenome	11,709	235,063,952	0:00:31.0 (32 chunks) ~ 0:00:52.1 (1 chunk)	0:05:57.3	0:03:12.7	0:03:26.9	0:00:14.8	0:00:02.2	0:00:00.8

This table provides characteristics and building (indexing) times of all reference sequences used in this paper. The left side of the table gives details on where the reference sequences were employed (section, subsection), the corresponding organism, the number of contigs, and the reference size in base pairs. The right side divides the tools into two categories and provides the time taken to build the index: FM-index (SigAlign, bowtie2, BWA-MEM, and HISAT2) and hashing (minimap2, BLASTn, and MMseqs2). The building time for each tool's reference structure is recorded in the format "hours:minutes:seconds".

Supplementary Table 7. Consistent Sensitivity Across Simulated Genomes

Cutoffs in SigAlign	Minimum Length	150	180	210	$2\sqrt{l}$	$5\sqrt{l}$	$8\sqrt{l}$
	**Maximum Penalty per Length**	**0.02**	**0.04**	**0.06**	$1.5/\sqrt{l}$	$0.5/\sqrt{l}$	$0.4/\sqrt{l}$
Common Name (Scientific Name)	Mtb (*M. tuberculosis*)	0.972815	0.996611	0.999484	1	0.999724	0.989135
	Ecoli (*E. coli*)	0.973454	0.996713	0.999495	1	0.999728	0.989459
	Yeast (*S. cerevisiae*)	0.973547	0.996635	0.999519	1	0.999710	0.989512
	ThaleCress (*A. thaliana*)	0.973109	0.996624	0.999500	1	0.999701	0.989234
	FruitFly (*D. melanogaster*)	0.973363	0.996690	0.999503	1	0.999724	0.989422
	Rice (*O. sativa*)	0.971762	0.996418	0.999461	1	0.999706	0.988626
	Zebrafish (*D. rerio*)	0.973757	0.996752	0.999515	1	0.999736	0.989612
	Mouse (*M. musculus*)	0.973815	0.996777	0.999520	1	0.999729	0.989627
	Human (*H. sapiens*)	0.973852	0.996797	0.999510	1	0.999762	0.989687

This table verifies that SigAlign maintains nearly constant sensitivity regardless of the origin of the reference used in simulation, given identical parameters. It demonstrates how sensitivity changes for each reference genome based on two of SigAlign's cutoffs: minimum length (MinL) and maximum penalty per length (MaxP). The first, second, and third columns for MinL and MaxP used fixed values. The fourth, fifth, and sixth columns expressed these parameters as functions of query length l, in the same manner as used in SigAlign's main paper. The parameters for the *lenient* setting correspond to the fourth column, and those for the *strict* setting correspond to the fifth column. The penalties for SigAlign were applied in accordance with the main paper, using values of 4, 6, and 2 for mismatch, gap-open, and gap-extend, respectively. The dataset included the same simulated reads discussed in the "Accuracy in Simulated Short Reads Across Organisms" subsection of the main paper.

Supplementary Table 8. Detailed Values of RMSE and MAE in Metagenome Abundance Profiling

Loos Functions	Databases	SigAlign (*strict*)	SigAlign (*lenient*)	BLASTn	MMseqs2	BWA-MEM	HISAT2	Bowtie2	Minimap2
**RMSE**	**Restricted**	60.82 (±0.0482)	250.54 (±0.0237)	441.19 (±0.0313)	1574.41 (±0.0310)	799.05	65.05	578.65	329.16
	**Full**	7.73 (±0.0277)	6.79 (±0.0285)	7.46 (±0.0266)	7.17 (±0.0318)	6.97	28.67	6.96	6.8
	**Extended**	9.56 (±0.0271)	8.54 (±0.0259)	9.19 (±0.0290)	10.11 (±0.0266)	8.86	30.3	8.45	8.78
**MAE**	**Restricted**	43.10 (±0.0251)	196.93 (±0.0273)	344.69 (±0.0308)	1165.80 (±0.0297)	590.86	52.78	439.7	244.08
	**Full**	3.70 (±0.0115)	2.63 (±0.0125)	3.31 (±0.0122)	3.02 (±0.0119)	2.62	22.7	2.74	2.59
	**Extended**	4.99 (±0.0103)	3.85 (±0.0102)	4.31 (±0.0118)	4.91 (±0.0136)	3.82	23.35	3.96	3.76

This table provides detailed values for Root Mean Square Error (RMSE) and Mean Absolute Error (MAE), both expressed in micro (µ) units, quantifying losses in relative abundance across various databases. The "Restricted" and "Full" database results are used in the main text, while the "Extended" database results are presented in Supplementary Figure 3. For SigAlign, BLASTn, and MMseqs2, the reported values are averages of 50 measurements with different random states, and the ± values represent the 99% confidence intervals (CI). RMSE is calculated as:

$$RMSE = \sqrt{\frac{1}{n}\sum_{i=1}^{n}(predicted_i - observed_i)^2}$$

and MAE as:

$$MAE = \frac{1}{n}\sum_{i=1}^{n}|predicted_i - observed_i|$$

where n denotes the number of genomes common to the query and reference, with values of 100 for the "Restricted" database and 200 for both the "Full" and "Extended" databases. $predicted_i$ represents predicted relative abundance (see Supplementary Note 14 for calculation details) and $observed_i$the actual relative abundance. In our tests, the queries were uniformly sampled across all genomes, thus the observed values are consistently 0.01 for the "Restricted" database and 0.005 for both the "Full" and "Extended" databases.

Supplementary Table 9. Detailed Values of False Negative Rate in Database Search Test

Num. Strains	Tools											
	SigAlign (*strict*)	SigAlign (*lenient*)	BLASTn	MMseqs 2	BWA-MEM	BWA-MEM (A)	Bowtie2	Bowtie2 (A)	HISAT2	HISAT2 (A)	Minimap 2	Minimap 2 (A)
**Initial Reference**												
1	0.0000E+00	0.0000E+00	0.0000E+00	0.0000E+00	0.0000E+00	0.0000E+00	0.0000E+00	0.0000E+00	0.0000E+00	0.0000E+00	0.0000E+00	0.0000E+00
2	0.0000E+00	0.0000E+00	0.0000E+00	3.5190E-03	3.7182E-01	2.1436E-02	3.7073E-01	0.0000E+00	5.4802E-02	2.5069E-02	2.0614E-03	2.0116E-03
4	0.0000E+00	0.0000E+00	0.0000E+00	9.9219E-03	7.4060E-01	5.5658E-02	7.3748E-01	3.4000E-05	3.5035E-01	6.6254E-02	4.0106E-02	3.9986E-02
8	0.0000E+00	0.0000E+00	0.0000E+00	1.5483E-02	8.8091E-01	1.0403E-01	8.7946E-01	9.4700E-05	6.1207E-01	1.1726E-01	1.9335E-01	1.4137E-01
16	0.0000E+00	0.0000E+00	0.0000E+00	2.2370E-02	9.1913E-01	1.2756E-01	9.1724E-01	1.1166E-04	7.0635E-01	1.5603E-01	5.2713E-01	1.7844E-01
32	0.0000E+00	0.0000E+00	0.0000E+00	2.9398E-02	9.6383E-01	4.6785E-01	9.6251E-01	1.2379E-04	8.4472E-01	3.1935E-01	7.4833E-01	2.4194E-01
64	0.0000E+00	0.0000E+00	0.0000E+00	3.8241E-02	9.8942E-01	6.4907E-01	9.8878E-01	1.0923E-04	9.3181E-01	4.6148E-01	8.9760E-01	2.6078E-01
128	0.0000E+00	0.0000E+00	0.0000E+00	4.8208E-02	9.9605E-01	7.7104E-01	9.9592E-01	9.7100E-05	9.7369E-01	6.0339E-01	9.5799E-01	3.2083E-01
256	0.0000E+00	0.0000E+00	0.0000E+00	5.6168E-02	9.9857E-01	8.1881E-01	9.9839E-01	9.9500E-05	9.8958E-01	7.0112E-01	9.8621E-01	3.3587E-01
512	0.0000E+00	0.0000E+00	0.0000E+00	8.0460E-02	9.9935E-01	8.5142E-01	9.9935E-01	1.1894E-04	9.9509E-01	7.5152E-01	9.9469E-01	3.4716E-01
1024	0.0000E+00	0.0000E+00	0.0000E+00	9.5752E-02	9.9968E-01	8.8913E-01	9.9972E-01	1.1166E-04	9.9791E-01	8.0570E-01	9.9771E-01	3.5886E-01
2048	0.0000E+00	0.0000E+00	0.0000E+00	1.0490E-01	9.9989E-01	9.3423E-01	9.9994E-01	1.0680E-04	9.9907E-01	8.6788E-01	9.9912E-01	3.9454E-01
4096	0.0000E+00	0.0000E+00	0.0000E+00		9.9995E-01	9.5846E-01	9.9994E-01		9.9960E-01	8.8335E-01	9.9957E-01	3.9535E-01
**Half-sized Reference**												
1	0.0000E+00	0.0000E+00	0.0000E+00	0.0000E+00	0.0000E+00	0.0000E+00	0.0000E+00	0.0000E+00	0.0000E+00	0.0000E+00	0.0000E+00	0.0000E+00
2	0.0000E+00	0.0000E+00	0.0000E+00	3.5190E-03	3.7182E-01	2.1436E-02	3.7073E-01	0.0000E+00	5.4802E-02	2.5069E-02	2.0614E-03	2.0116E-03
4	0.0000E+00	0.0000E+00	0.0000E+00	6.6001E-03	6.7885E-01	3.8216E-02	7.3436E-01	4.0200E-05	3.3189E-01	6.5126E-02	3.9690E-02	3.9697E-02
8	0.0000E+00	0.0000E+00	0.0000E+00	5.4934E-03	6.6182E-01	6.1744E-02	6.7538E-01	6.1700E-05	4.2644E-01	9.2402E-02	1.7709E-01	1.0535E-01
16	0.0000E+00	0.0000E+00	3.1800E-07	8.3919E-03	6.3811E-01	3.5260E-02	5.9798E-01	6.4700E-05	3.5490E-01	1.2673E-01	4.2648E-01	4.4003E-02
32	0.0000E+00	0.0000E+00	0.0000E+00	8.3772E-03	7.0801E-01	4.1259E-01	7.1257E-01	7.3300E-05	5.4635E-01	2.7828E-01	5.1671E-01	8.2623E-02
64	0.0000E+00	0.0000E+00	0.0000E+00	1.0353E-02	5.9704E-01	3.7017E-01	5.9993E-01	5.7500E-05	5.1755E-01	2.6026E-01	5.1305E-01	2.6389E-02
128	0.0000E+00	0.0000E+00	0.0000E+00	1.1386E-02	7.8245E-01	3.9799E-01	7.8606E-01	5.3300E-05	6.9429E-01	3.4684E-01	6.1523E-01	8.7952E-02

256	0.0000E+00	0.0000E+00	7.6300E-08	9.8339E-03	7.2184E-01	2.2978E-01	7.3353E-01	5.1000E-05	5.8008E-01	3.1682E-01	6.2773E-01	2.4116E-02
512	0.0000E+00	0.0000E+00	0.0000E+00	2.7913E-02	7.4199E-01	2.1611E-01	7.5797E-01	5.0700E-05	5.8569E-01	2.5347E-01	6.0441E-01	1.8669E-02
1024	0.0000E+00	0.0000E+00	9.3800E-09	1.8694E-02	8.4304E-01	2.8388E-01	8.5055E-01	5.2800E-05	7.0401E-01	2.8468E-01	7.0060E-01	1.7377E-02
2048	0.0000E+00	0.0000E+00	0.0000E+00	1.1872E-02	9.1149E-01	4.2795E-01	9.1730E-01	5.3500E-05	8.1443E-01	3.6883E-01	7.5598E-01	5.7831E-02
4096	0.0000E+00	0.0000E+00	1.3300E-07		7.0452E-01	3.7184E-01	7.0518E-01		7.6176E-01	1.7259E-01	7.7059E-01	2.3045E-03

This table presents the False Negative Rate (FNR) values as discussed in the "Recall of Alignment Results in Large Database" (of “Application in Database Search” section) of the main paper. It details FNR for both 1) the initial reference and 2) the half-sized reference. The initial reference encompasses only a single strain, whereas the half-sized reference comprises half the strains of the tested reference. For instance, if the tested reference has 512 strains, the half-sized reference contains 256 strains. Tools marked with "(A)" used an option to display all results, whereas those without "(A)" relied on their default settings. For specific commands, refer to Supplementary Table 3.

Supplementary Table 10. Reasons Behind the Reduction in Mean Memory Usage by Additional Threads

Parameter Sets	Threads	Total Time (s)	Reference Loading Time (s)	Ratio of Reference Loading (%)	Max Mem Usage (MiB)	Mean Mem Usage (MiB)
Strict	1	68.99	0.93	1.34	1,245.80	1,232.43
	2	41.08	0.93	2.26	1,246.25	1,226.76
	4	23.25	0.93	4.01	1,246.34	1,214.20
	8	12.53	0.93	7.40	1,247.01	1,190.99
	16	7.07	0.93	13.09	1,248.05	1,151.76
Lenient	1	166.26	0.93	0.56	1,246.75	1,238.34
	2	98.63	0.93	0.94	1,247.22	1,236.43
	4	52.57	0.93	1.78	1,248.31	1,231.41
	8	29.70	0.93	3.14	1,249.68	1,222.57
	16	18.88	0.91	4.84	1,252.16	1,212.58

The table delineates alignment time, reference loading duration, and both max and mean memory usage for each parameter set (*strict* and *lenient*) of the SigAlign in relation to increasing thread count. Notably, while reference loading time remains fairly consistent, alignment time is reduced with the augmentation of threads. Memory usage begins from a baseline of 0 and rises progressively as the reference is loaded. Given that the reference constitutes the majority of total memory usage and the space reserved for the alignment process is minimal (refer to the "Memory Footprint" section in the main paper), the memory allocated during the reference loading phase approximates to half of that designated for the alignment phase. Consequently, with an escalation in thread count, the fraction of total time dedicated to reference loading amplifies, whereas the mean memory usage exhibits a decline.

Supplementary Table 11. Performance of RazerS3 for Read Mapping Tasks

(a) Performance for Diverse Sequencing Platforms			
**Data**	**Throughput (read/s)**	**Identity (%)**	**Mapping rate (%)**
Illumina NovaSeq	69,220	99.82	90.32
Illumina MiSeq	16,858	99.05	89.74
Nanopore MinION (R10.4)	0.158		0
PacBio Sequel (HiFi)	0.0084	100	0.1

(b) Accuracy in Simulated Short Reads Across Organisms				
**Data**	**Sensitivity**	**Precision**	**Alignment**	**Throughput (read/s)**
Mycobacterium tuberculosis	1	0.927377	1.08	21,396.74
Escherichia coli	1	0.804823	1.24	16,652.17
Saccharomyces cerevisiae	1	0.604031	1.66	7,616.03
Arabidopsis thaliana	1	0.634416	1.58	1,715.44
Drosophila melanogaster	1	0.161246	6.28	637.97
Oryza sativa	1	0.065138	15.40	203.69
Danio rerio	1	0.019019	53.74	2.40
Mus musculus	1	0.002833	357.36	4.89
Homo sapiens	1	0.003877	258.53	5.00

This table showcases the performance of RazerS3 (version 3.5.8) in read mapping tasks as outlined in the "Read Mapping Across Various Sequencing Data" section of the main text, divided into tests (**a**) and (**b**). Except for specific datasets, RazerS3 utilized a 1/100 subsampled query for speed, excluding the following data: Illumina NovaSeq and Illumina MiSeq data in (a), and "*Mycobacterium tuberculosis*" and "*Escherichia coli*" data in (b). The commands used included RazerS3's default options with the addition of setting the "--max-hits" parameter to 10 million to output all alignment positions. The reasons for RazerS3's exclusion from the tools tested, due to certain differences, are detailed in the "Other Aligners Beyond the Test Settings" section of the main paper's Discussion. The results demonstrate RazerS3's ability to achieve high speeds with small and less complex references (e.g., bacterial genomes) and short reads in (a), showcasing its advantage of perfect sensitivity in the simulated tests with short reads in (b). However, similar to SigAlign and database search tools, RazerS3 exhibits a disadvantage of low precision in simulated test (b). Additionally, its limitation to only perform

end-to-end alignments resulted in no alignment findings for MinION data in the various platform tests, and only 0.1% of reads could be mapped in PacBio data (a).

Supplementary Table 12. Precision in Simulated Short Reads Using Primary Alignment

Reference	SigAlign (*strict*)	SigAlign (*lenient*)	Bowtie2	BWA-MEM	HISAT2	Minimap2	BLASTn	MMseqs2
**TB**	0.9943 (2)	0.9945 (1)	0.9924 (5)	0.9924 (4)	0.9901 (8)	0.9923 (6)	0.9935 (3)	0.9903 (7)
**Ecoli**	0.9804 (2)	0.9810 (1)	0.9748 (4)	0.9747 (5)	0.9718 (8)	0.9747 (6)	0.9779 (3)	0.9730 (7)
**Yeast**	0.9754 (2)	0.9759 (1)	0.9744 (4)	0.9744 (5)	0.9727 (7)	0.9741 (6)	0.9751 (3)	0.9725 (8)
**ThaleCress**	0.9919 (2)	0.9921 (1)	0.9887 (5)	0.9887 (4)	0.9859 (7)	0.9886 (6)	0.9905 (3)	0.9795 (8)
**FruitFly**	0.9356 (4)	0.9370 (2)	0.9354 (5)	0.9357 (3)	0.9349 (6)	0.9345 (7)	0.9415 (1)	0.9239 (8)
**Rice**	0.9767 (2)	0.9782 (1)	0.9729 (5)	0.9737 (4)	0.9690 (7)	0.9727 (6)	0.9760 (3)	0.9619 (8)
**Zebrafish**	0.8903 (2)	0.8935 (1)	0.8879 (5)	0.8899 (3)	0.8849 (7)	0.8888 (4)	0.8855 (6)	0.8642 (8)
**Mouse**	0.9647 (2)	0.9674 (1)	0.9545 (5)	0.9568 (4)	0.9520 (7)	0.9483 (8)	0.9640 (3)	0.9523 (6)
**Human**	0.9672 (2)	0.9675 (1)	0.9449 (5)	0.9466 (4)	0.9414 (6)	0.9396 (7)	0.9618 (3)	0.9202 (8)

In the manuscript's section "Accuracy in Simulated Short Reads Across Organisms," we define precision as the proportion of alignments correctly positioned out of the total number of alignments. This approach reveals that both SigAlign and database search tools (BLASTn, MMseqs2) experience a significant drop in precision with an increase in the number of alignment locations (as shown in Figure 2b). However, similar to read mappers, selecting a "primary alignment" for each query—essentially the best alignment—can enhance precision for both SigAlign and the database search tools. The criteria for selecting the "primary alignment" for each tool were as follows:

- SigAlign: The alignment covering the longest portion of the query, with ties broken by choosing the alignment with the smaller penalty.
- BLASTn, MMseqs2: The alignment with the smallest E-value.
- Read mappers: Alignments designated as "primary alignment" according to the SAM format.

The above criteria are the same as those used for selecting a single alignment in the "Human Gut Metagenome Abundance Profiling" section of the main text. Specifically, in cases of ties, a random selection was made for SigAlign, BLASTn, and MMseqs2 (for detailed methods on random selection, see "Supplementary Note 14. Calculations for Relative Abundance"). However, only a seed value of "0"

was used for numpy's "seed," and for SigAlign's results on Zebrafish, the data was combined from 10 and 5 separate chunks for *strict* and *lenient* settings, respectively, due to the large file sizes (180GB and 90GB, respectively).

The values in the table represent the precision calculated based on these "primary alignments," rounded to four decimal places. The numbers in parentheses indicate the rank of precision for each reference genome among the tools evaluated. SigAlign demonstrated the highest precision in nearly all cases when using the *lenient* parameter setting, with the exception of its performance on the FruitFly genome compared to BLASTn. Furthermore, with the *strict* parameter setting, SigAlign exhibited the second-highest precision across all organisms except for FruitFly.

Supplementary Figures

Supplementary Figure 1. Iteration of Minimum Penalty Across Consecutive K-mers

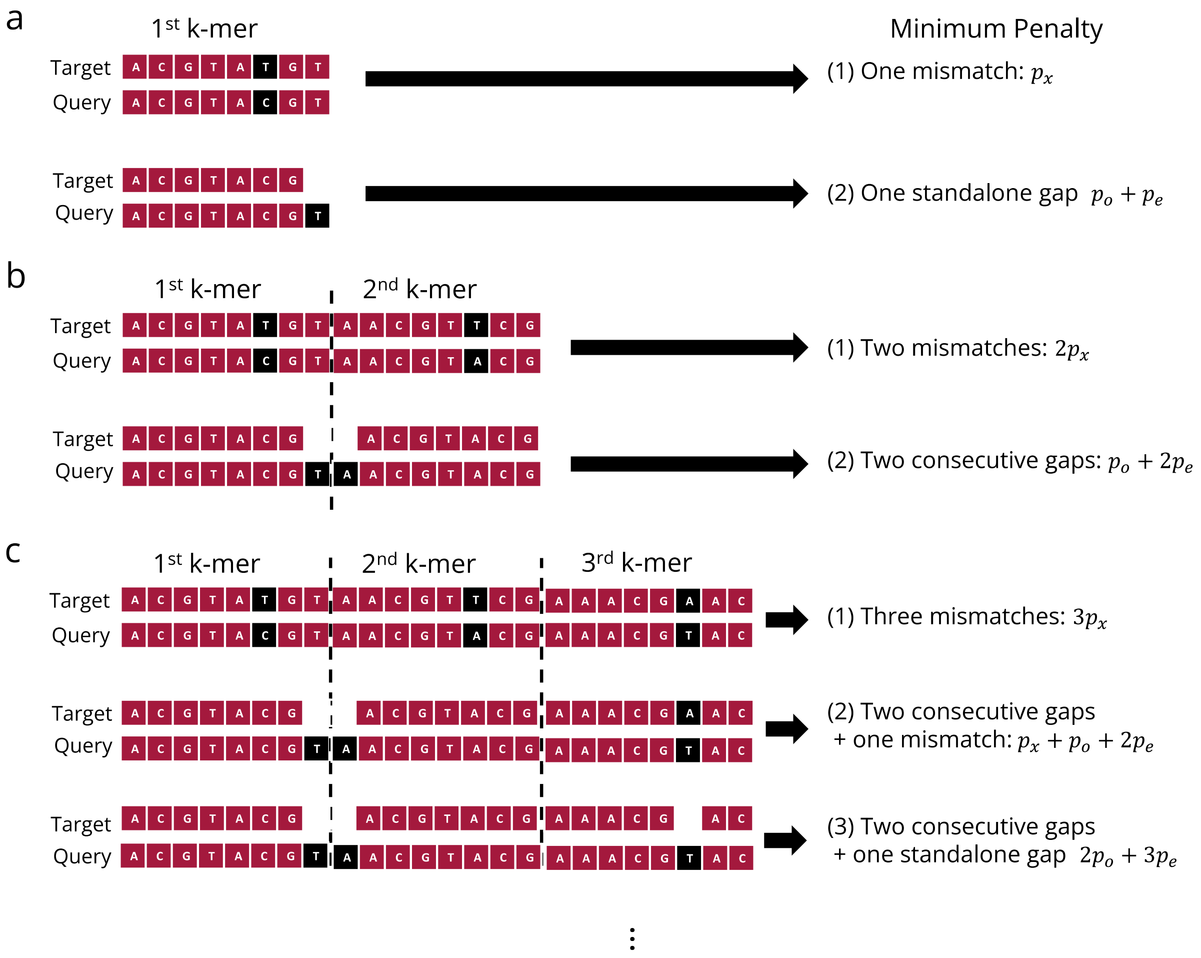

This figure provides a detailed representation of the minimum penalty associated with consecutive k-mers that remain unmatched. In panel (**a**), a scenario with a single unmatched k-mer is presented. The minimum penalty in this situation emerges from either a singular mismatch or a standalone gap, contingent on the gap-affine penalty value. Panel (**b**) portrays a scenario with two successive unmatched k-mers. Here, the minimum penalty originates from either dual mismatches or two sequential gaps. Panel (**c**) displays a case where three successive k-mers are unmatched. It's noteworthy that the value in (**c**) can be confirmed to equate to the aggregate of values in (**a**) and (**b**). Hence, a formulation to determine the minimum penalty based on the number of k-mers becomes feasible. The standard equation corresponding to this finding is elaborated upon in the main paper, delineated as the formula for $\hat{p}$.

Supplementary Figure 2. Relationship between $\hat{l}_n$ and $\hat{p}_n$

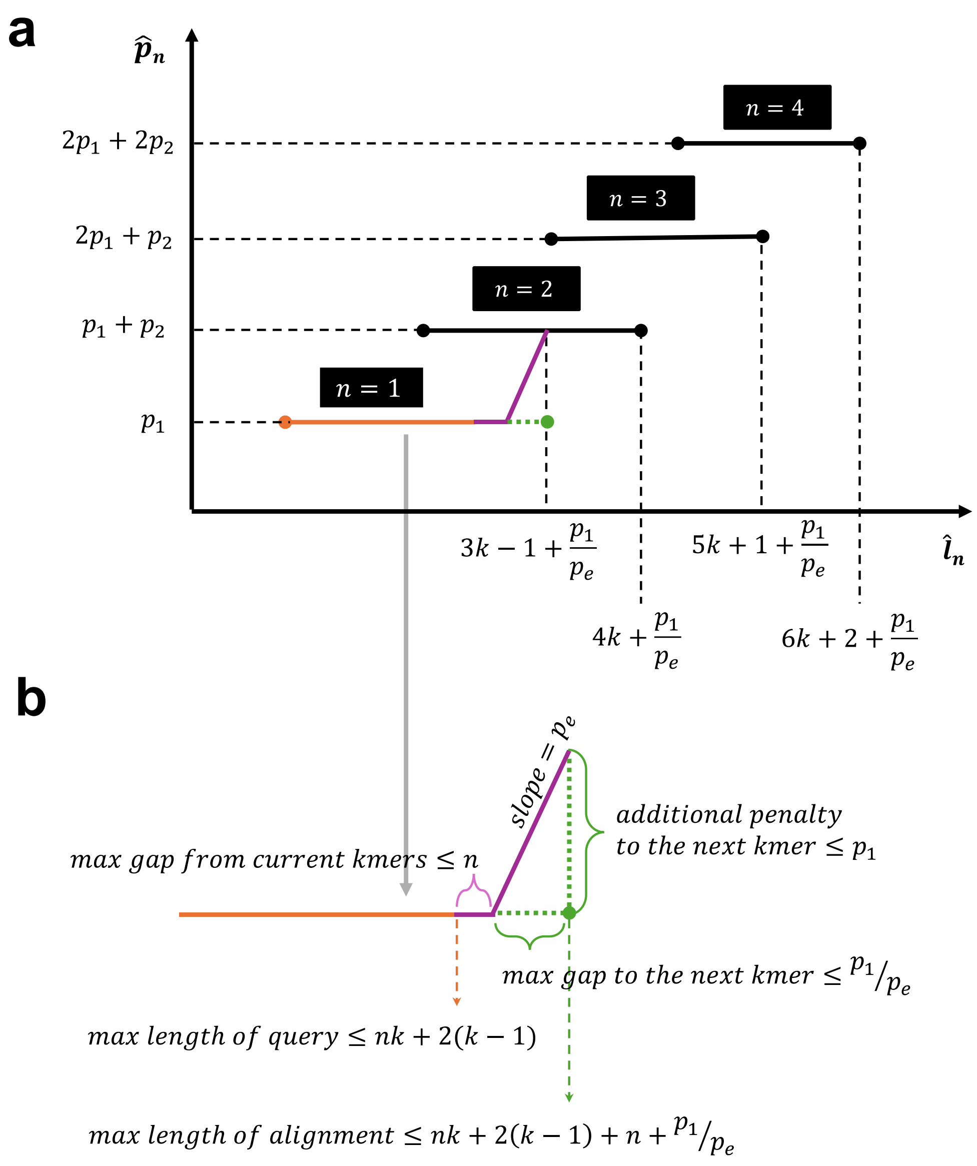

This graph illustrates the relationship between $\hat{l}_n$ (the length of an alignment that can be represented by n k-mers) and $\hat{p}_n$ (the penalty for alignments where none of the n k-mers match). (**a**) depicts the relationship graph between the two variables. As shown, the length range of an alignment that can be represented by n k-mers overlaps with that for $n + 1$ k-mers. Since we are interested in the minimum penalty per length ($\hat{d}_n$), it is crucial to indicate the range for the maximum $\hat{l}_n$ for each n. For $(n = 1)$ as an example, we further dissected the relationship between length and penalty, distinguishing with colors. (**b**) provides descriptions for the color-differentiated lines. The orange line represents the case without gaps, indicating the length of the query. Here, the minimum penalty corresponds with $\hat{p}_n$. The purple line represents cases

with gaps. If gaps occur in the current k-mers, there can be up to n gaps, and the minimum penalty still corresponds with $\hat{p}_n$. However, with more than n gaps, the minimum penalty increases by p_e per gap from $\hat{p}_n$. The lime dotted line simplifies the calculation for efficiency, using the value at the point where the penalty for using n k-mers equals the minimum penalty for $n + 1$ k-mers, thus serving as the maximum alignment length that can be represented by n k-mers, when penalty=$\hat{p}_n$.

Supplementary Figure 3. Metagenome Profiling Results for the Extended Database

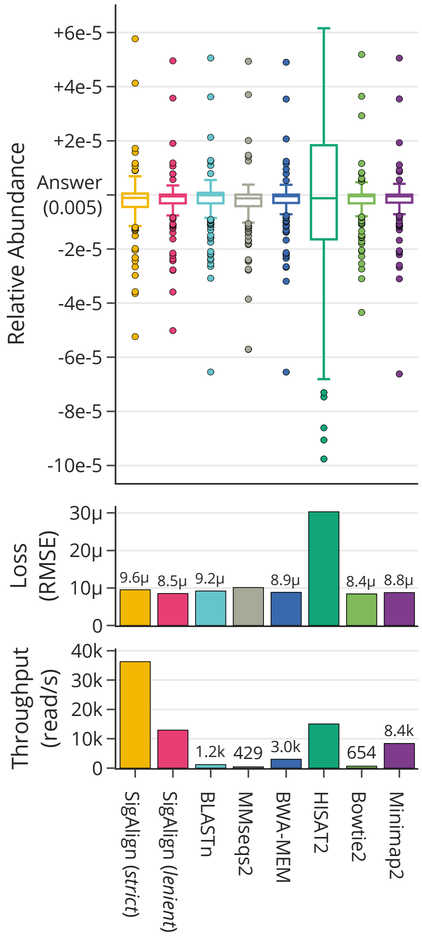

This figure presents the results of metagenome profiling for the "Extended" database, which includes the top 400 genera, following the same analysis performed for the "Human Gut Metagenome Abundance Profiling" section and Figure 3 in the main text. The format of this figure matches that of Figure 3: the top panel shows the relative abundance distributions, the middle panel shows the loss measured by RMSE (Root Mean Square Error), and the bottom panel shows the throughput.

In the Extended database, there was a slight decrease in speed and an increase in RMSE compared to the Full database. However, the comparative performance of the tools and the patterns of the distributions remained consistent with those observed for the Full database. Therefore, the implications of the Extended database results are similar to those of the Full database, which is why these results are included in the supplementary material rather than the main text.

Supplementary Figure 4. Database Search Results for Read-Mappers

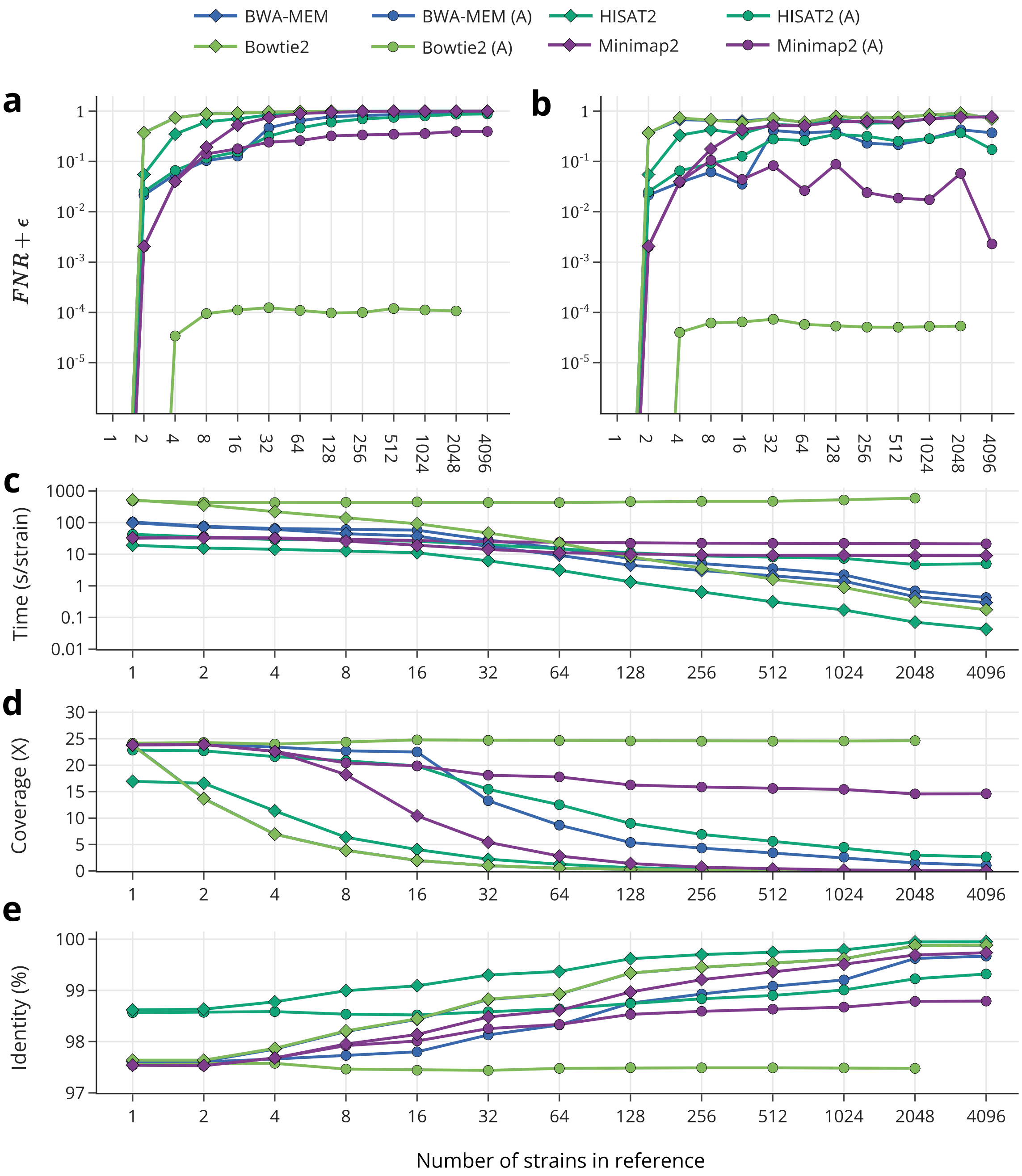

This figure delineates the performance metrics from Figure 4 under the "Recall of Alignment Results in Large Database" subsection (in "Application in Database Search"), comparing read mappers under both their default settings and the option to display all results. Tools marked with "(A)" utilize the option to display all results. Refer to Supplementary Table 3 for the specific commands employed. The x-axis showcases the number of strains in the reference and is

displayed on a logarithmic scale. Graphs (**a**) and (**b**) chart the False Negative Rate (FNR) augmented by epsilon, as in Figure 4. These metrics are based on results from the first reference with a single strain (**a**) and the reference with half the strains (**b**). Graph (**c**) conveys the processing time (measured in seconds) normalized by the number of strains. Graph (**d**) highlights coverage, quantified as the ratio of total output length to reference length. Lastly, (**e**) portrays percent identity, defined as the ratio of matched base pairs to total output length. The y-axis for graphs (**a**), (**b**), and (**c**) is logarithmic. Overall, this figure underscores the performance disparities between SigAlign, database search tools and read mappers.

Supplementary Figure 5. Time Series Memory Usage for All Tools

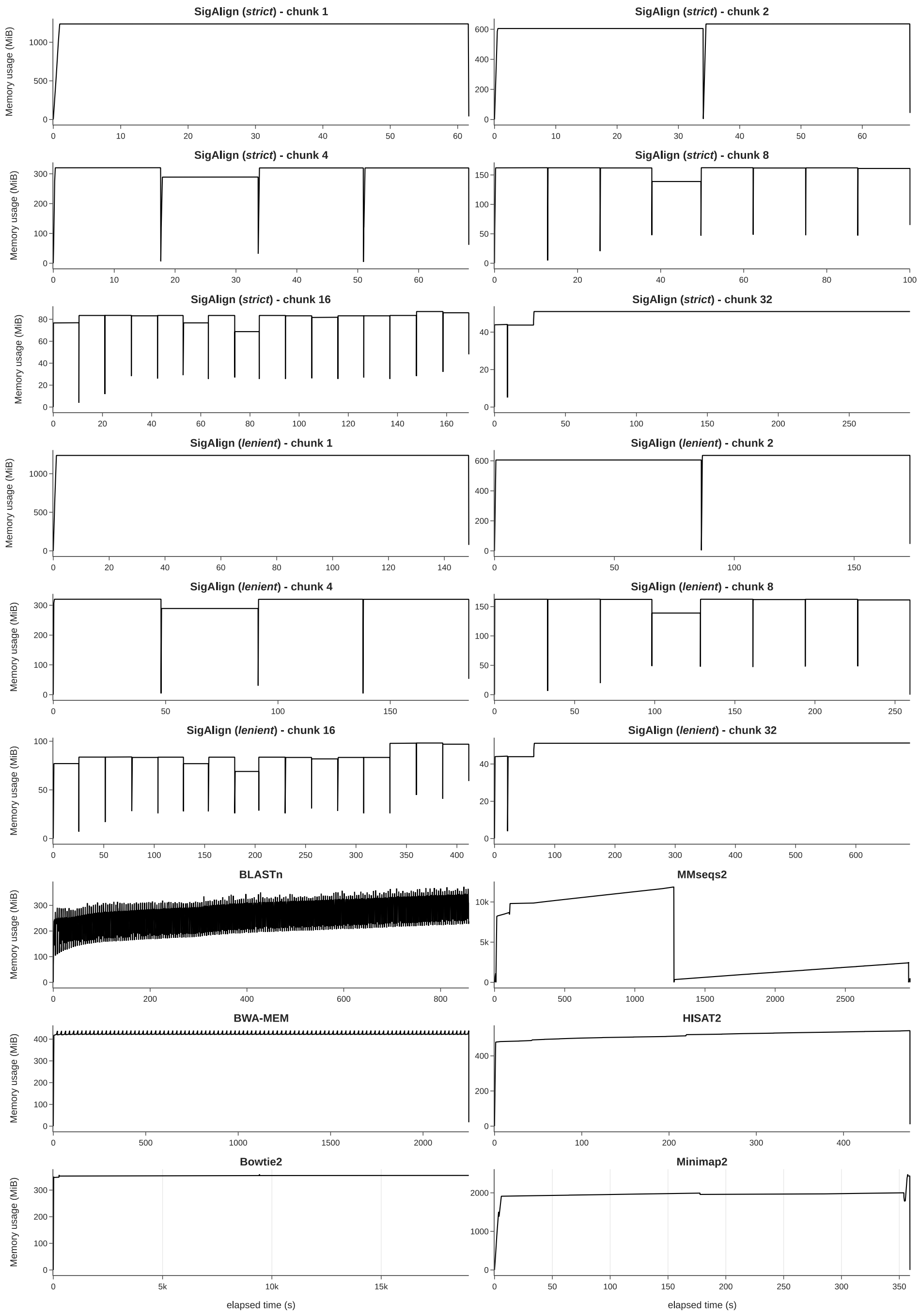

This graph represents memory utilization as a function of time, utilizing data from the memory usage tests pertaining to different SigAlign chunks, as discussed in the main text. This depiction offers an alternative view of the results presented in Figure 5a.

Supplementary Figure 6. Average Memory Usage Across Various Query Lengths

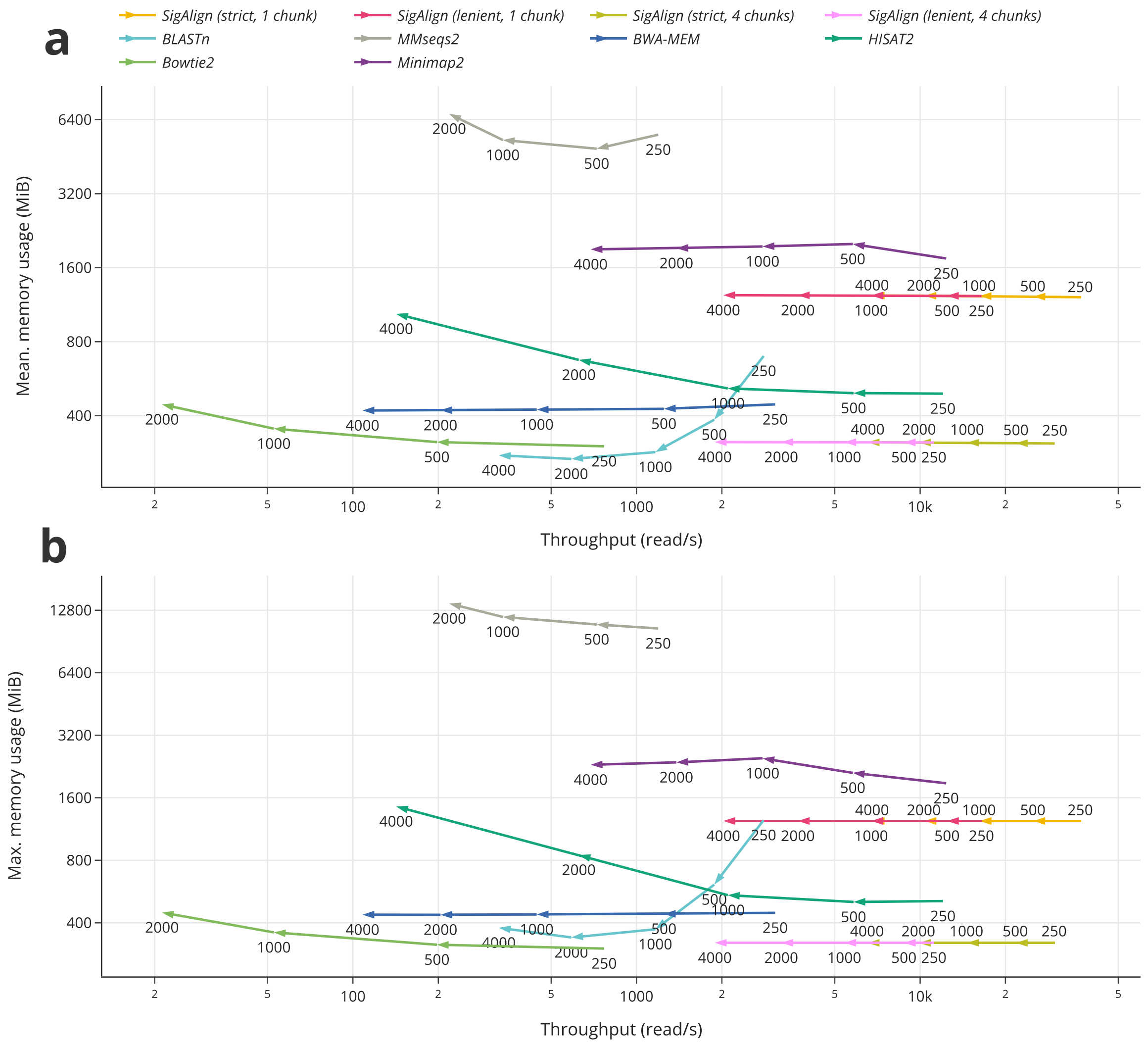

This graph presents the results of evaluating memory usage across different query lengths not only for SigAlign but also for other tools. (a) depicts the average memory usage on the y-axis, while (b) showcases the maximum memory usage, with throughput consistently on the x-axes. The graphs use arrows as symbols to demonstrate the performance changes sequentially from short to long lengths (250 -> 500 -> 1000 -> 2000 -> 4000 bp). These graphs allow us to observe that the fluctuation in memory usage in SigAlign is minimal compared to other tools. More specifically, the differences between the maximum and minimum values of average and maximum memory usage for each tool are as follows (measured in MiB):

- SigAlign (*strict*, 1 chunk): gap for average 19.82, gap for maximum 0.18
- SigAlign (*lenient*, 1 chunk): gap for average 9.18, gap for maximum 0.20
- SigAlign (*strict*, 4 chunks): gap for average 3.23, gap for maximum 0.18

- SigAlign (*lenient*, 4 chunks): gap for average 1.03, gap for maximum 0.21
- BLASTn: gap for average 432.63, gap for maximum 908.52
- MMseqs2: gap for average 1882.91, gap for maximum 3344.58
- Bowtie2: gap for average 144.26, gap for maximum 147.61
- BWA-MEM: gap for average 24.47, gap for maximum 9.46
- HISAT2: gap for average 546.21, gap for maximum 949.20
- Minimap2: gap for average 253.45, gap for maximum 598.75

References

1. Needleman,S.B. and Wunsch,C.D. (1970) A general method applicable to the search for similarities in the amino acid sequence of two proteins. *J Mol Biol*, **48**, 443–53.
2. Smith,T.F. and Waterman,M.S. (1981) Identification of common molecular subsequences. *J Mol Biol*, **147**, 195–7.
3. Gotoh,O. (1982) An improved algorithm for matching biological sequences. *J Mol Biol*, **162**, 705–8.
4. Schbath,S., Martin,V., Zytnicki,M., Fayolle,J., Loux,V. and Gibrat,J.-F. (2012) Mapping Reads on a Genomic Sequence: An Algorithmic Overview and a Practical Comparative Analysis. *J. Comput. Biol.*, **19**, 796–813.
5. Burrows,M. (1994) A block-sorting lossless data compression algorithm. *SRS Res. Rep.*, **124**.
6. Anderson,T. and Wheeler,T.J. (2021) An optimized FM-index library for nucleotide and amino acid search. *Algorithms Mol. Biol.*, **16**.
7. Marco-Sola,S., Moure,J.C., Moreto,M. and Espinosa,A. (2021) Fast gap-affine pairwise alignment using the wavefront algorithm. *Bioinformatics*, **37**, 456–463.
8. Meehan,C.J., Goig,G.A., Kohl,T.A., Verboven,L., Dippenaar,A., Ezewudo,M., Farhat,M.R., Guthrie,J.L., Laukens,K., Miotto,P., *et al.* (2019) Whole genome sequencing of Mycobacterium tuberculosis: current standards and open issues. *Nat. Rev. Microbiol.*, **17**, 533–545.
9. Ni,Y., Liu,X., Simeneh,Z.M., Yang,M. and Li,R. (2023) Benchmarking of Nanopore R10.4 and R9.4.1 flow cells in single-cell whole-genome amplification and whole-genome shotgun sequencing. *Comput. Struct. Biotechnol. J.*, **21**, 2352–2364.
10. Chen,S. (2023) Ultrafast one-pass FASTQ data preprocessing, quality control, and deduplication using fastp. *iMeta*, **2**.
11. Almeida,A., Nayfach,S., Boland,M., Strozzi,F., Beracochea,M., Shi,Z.J., Pollard,K.S., Sakharova,E., Parks,D.H., Hugenholtz,P., *et al.* (2021) A unified catalog of 204,938 reference genomes from the human gut microbiome. *Nat. Biotechnol.*, **39**, 105–114.
12. Hiseni,P., Rudi,K., Wilson,R.C., Hegge,F.T. and Snipen,L. (2021) HumGut: a comprehensive human gut prokaryotic genomes collection filtered by metagenome data. *Microbiome*, **9**, 165.
13. Holtgrewe,M. (2010) Mason–a read simulator for second generation sequencing data. *Tech. Rep. FU Berl.*
14. Milhaven,M. and Pfeifer,S.P. (2023) Performance evaluation of six popular short-read simulators. *Heredity*, **130**, 55–63.
15. Gourlé,H., Karlsson-Lindsjö,O., Hayer,J. and Bongcam-Rudloff,E. (2019) Simulating Illumina metagenomic data with InSilicoSeq. *Bioinformatics*, **35**, 521–522.
